# *In situ* extended immune activation instantly after tumor resection by oncolytic virus controls postoperative tumor recurrence

**DOI:** 10.1016/j.xcrm.2025.102399

**Published:** 2025-10-03

**Authors:** Ciliang Guo, Tian Gao, Bin Xue, Louqian Zhang, Shuo Wang, Rongrong Xiao, Lingkai Kong, Yuxin Zhang, Qilei Xin, Yi Cao, Xiaosong Gu, Chunping Jiang, Junhua Wu

**Affiliations:** 1 State Key Laboratory of Pharmaceutical Biotechnology, Department of General Surgery Nanjing Drum Tower Hospital, The Affiliated Hospital of Medical School, Medical School, Nanjing University, Nanjing 210008, China; 2 Jinan Microecological Biomedicine Shandong Laboratory, Building 1, Jinan Medical and Health Science and Technology Innovation Industrial Park, No. 288, Jiqi Road, Huayin District, Jinan City, Shandong Province, China; 3 “Nanjing University-Gulou” Joint Laboratory of AI and Healthcare BigData, National Institute of Healthcare Data Science at Nanjing University, School of Life Sciences, Jiangsu Key Laboratory of Molecular Medicine, Nanjing University, Nanjing 210093, China; 4 Collaborative Innovation Center of Advanced Microstructures, National Laboratory of Solid State Microstructure, Department of Physics, Nanjing University, Nanjing 210093, China; 5 Department of Hepatobiliary and Pancreatic Surgery, The Second Affiliated Hospital of Fujian Medical University, Quanzhou, Fujian Province 362000, China; 6 Renhuai People’s Hospital, Renhuai, Guizhou Province 564055, China

**Keywords:** tumor recurrence, oncolytic virus, hydrogel, tumor immunotherapy, immune activation, type I interferon pathway, anti-tumor immunity, oncolytic adenovirus, supramolecular hydrogel, sustained release

## Abstract

Postoperative tumor recurrence represents a major challenge for patients. Oncolytic virus (OV) therapy has attracted increasing attention in recent years. Here, we construct a supramolecular hydrogel enabling extended release of type V oncolytic adenovirus (adv), with hydrogel stability confirmed experimentally. *In situ* treatment with the adv-loaded hydrogel (adv@Nap gel) instantly after tumor resection efficiently activates the type I interferon pathway, induces innate and adaptive immunity, controls postoperative tumor recurrence and metastasis, and prolongs mouse survival. We verify the ability of instant *in situ* treatment with adv@Nap gel to inhibit postoperative tumor recurrence. Notably, oncolytic herpes simplex virus or vaccinia virus loaded in Nap gel can also control postoperative tumor recurrence. Thus, hydrogel-loaded OVs that induce extended immune activation represent a paradigm for sustained antitumor immunotherapy, and *in situ* sustained immune activation initiated during surgery may represent an important and universal treatment guideline.

## Highlights

• A stable hydrogel for sustained release of oncolytic viruses (OVs) is constructed
• *In situ* OVs@Nap gel treatment instantly after surgery controls tumor recurrence
• Adv@Nap gel treatment induces immune response via activating type I interferon pathway
• This strategy has broad-spectrum applicability across multiple oncolytic virus types

## Graphical abstract

**Figure:**
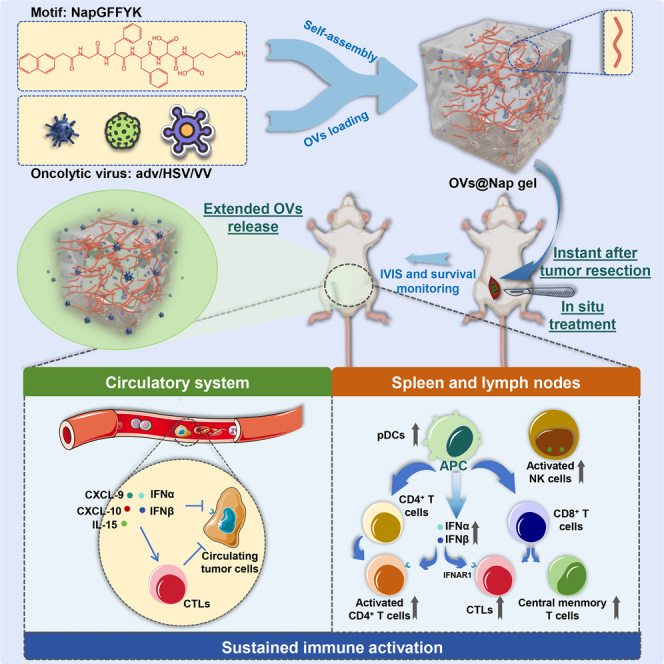

## Teaser

Guo et al. report that a supramolecular hydrogel enables sustained oncolytic virus (OV) release. Administering this OV-loaded hydrogel instantly after tumor resection induces prolonged immune activation, effectively curbing postoperative tumor recurrence and metastasis and increasing survival in mice. This strategy represents a universal and clinically translatable approach for cancer immunotherapy.

## Introduction

Tumors are major diseases that threaten human health and life.^1^ Surgical resection of the tumor mass is often the first choice for patients who have the opportunity for surgery. However, for patients who have undergone surgery, postoperative recurrence (*in situ* and metastatic recurrence) represents one of the greatest challenges that needs to be addressed.^2^ The recurrence time is also indefinite, and some cases of metastatic recurrence may occur years or even decades after surgery.^3^^,^^4^ Moreover, the wound healing process and associated inflammation lead to postoperative tumor recurrence.^5^ At present, chemotherapy and radiotherapy are commonly used to control postoperative recurrence in clinical practice. However, these methods often have strong toxic side effects in some cases.^6^^,^^7^ Therefore, there is an urgent need for safe and effective treatments to control tumor recurrence after surgery.

With respect to the reasons for tumor recurrence after surgery, owing to the complexity of the tumor itself, tumors may exhibit local tumor microinfiltration, and circulating tumor cells may be present; these factors can induce future tumor recurrence,^8^^,^^9^^,^^10^ including *in situ* recurrence and metastasis. Specifically, local tumor microinfiltration and circulating tumor cells not only suppress the innate immune response in various ways but also facilitate tumor cell escape from infiltrated immune effector cells.^11^^,^^12^^,^^13^ Furthermore, the wound healing process and associated inflammation can lead to an immunosuppressive microenvironment in the local area of surgery, followed by a systemic immunosuppressive state, helping tumor cells achieve immune escape and waiting for recurrence.^8^^,^^9^^,^^10^ Rapid advances in cancer immunotherapy, driven by promising clinical results and new drug approvals, offer new hope to cancer patients.^14^^,^^15^^,^^16^ Among these methods or drugs, some can eliminate the immune escape of tumor cells and directly induce antitumor immunity; some of these approaches have potential value in controlling postoperative tumor recurrence and metastasis.^17^^,^^18^^,^^19^^,^^20^ Therefore, tumor immunotherapy should have great potential application value in preventing postoperative recurrence.

For dosing modalities, the systemic administration of immunotherapies, such as immune checkpoint inhibitors,^21^^,^^22^ CAR (chimeric antigen receptor)-T cells,^23^^,^^24^ or CAR-natural killer (NK) cells,^25^ is mostly used to achieve long-term effective immune surveillance throughout the body. Studies have shown that intratumoral immunotherapy is safer and sometimes more effective than systemic treatment.^26^^,^^27^ Local concentrated treatment at the tumor site can disrupt local immune tolerance and induce systemic antitumor immunity while avoiding severe side effects.^26^^,^^28^ Thus, *in situ* immune activation is a promising strategy for tumor immunotherapy and prevention of tumor recurrence after surgery. Another important point is that wound healing and inflammation begin the moment the tumor is removed^29^; thus, to effectively control postoperative recurrence, immune activation should be induced as early as possible (i.e., instantly after tumor resection). In addition, postoperative wound healing is not a short process; thus, *in situ* immune regulation instantly after surgery should preferably be implemented once and maintained for a certain period, as repeated induction may cause new injuries.

Oncolytic virus (OV) therapy, a promising branch of cancer immunotherapy,^30^^,^^31^ utilizes viruses with antitumor effects that selectively replicate in and destroy cancerous tissues without damaging normal tissues.^30^^,^^32^^,^^33^ In addition to their direct oncolytic effect on tumor cells, OVs are capable of inducing systemic antitumor immune responses, which can transform “cold” tumors into “hot” ones. This outcome increases the susceptibility of tumors to other treatment modalities, thereby providing a practical opportunity for synergistic anticancer strategies.^34^^,^^35^^,^^36^^,^^37^ Among all types of OVs, adenovirus (adv), herpes simplex virus (HSV), and vaccinia virus (VV) are three OVs that have been commonly investigated in clinical trials over the last decade,^38^ indicating their potential for future application, although efficacy or safety challenges remain.^39^^,^^40^ Promisingly, biomaterials with novel drug delivery properties have facilitated remarkable progress in cancer immunotherapy.^41^^,^^42^^,^^43^ In recent years, as multipurpose biomaterials, hydrogels have been widely used in drug delivery, including for locally focused drug release,^44^ continuous drug release,^45^ and responsive drug release.^46^^,^^47^ Owing to their biodegradability and ability to persist stably within the body for a period, hydrogels are particularly well suited for immune regulation.^48^^,^^49^ Some hydrogels have been utilized as carriers for immunomodulatory drugs that target tumor cells to prevent recurrence and metastasis.^50^^,^^51^^,^^52^ In summary, these groundbreaking works on hydrogels have given us a lot of inspiration for tumor prevention and treatment.^44^^,^^45^^,^^46^^,^^47^^,^^48^^,^^49^^,^^50^^,^^51^^,^^52^

In this study, a supramolecular hydrogel (adv@Nap gel) was designed and constructed to achieve extended release of type Ⅴ oncolytic adv. The inhibition of tumor recurrence was assessed in an orthotopic mouse model of breast cancer treated via *in situ* placement of adv@Nap gel instantly after tumor resection, and the antitumor immune response was analyzed. Specifically, the type I interferon pathway and innate and adaptive immunity are activated during this process. Furthermore, the necessity of extended immune activation and *in situ* and instant treatment was confirmed. Additionally, we broadened our concept to include other types of OVs and validated our results in a humanized mouse tumor model. These findings provide an important treatment strategy and theoretical foundation for the development of methods to control postoperative tumor recurrence; importantly, the approach developed in this study has strong prospects for clinical translation.

## Results

### Oncolytic adv significantly activates the antitumor immune response

**Figure 1:**
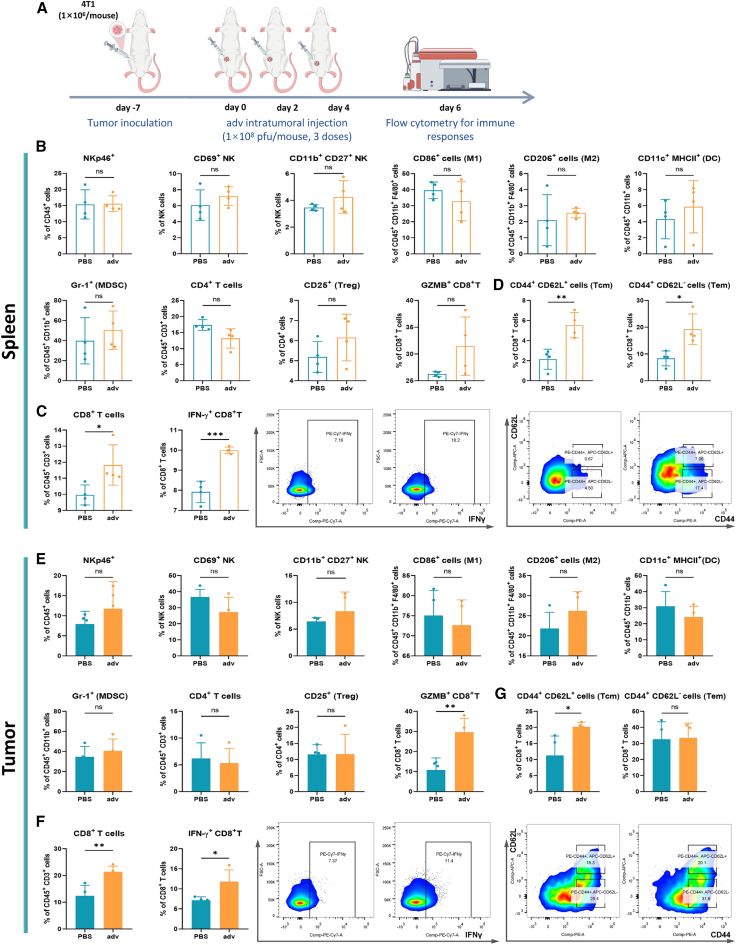
Antitumor immunomodulatory effects of oncolytic adenovirus treatment in an *in situ* breast cancer model (A) Schematic illustration of immune activation by adenovirus (adv) in a mouse model of *in situ* breast cancer. (B) Flow cytometric analysis of NK cells (NKp46^+^), activated NK cells (CD69^+^), high effector NK cells (CD11b^+^ CD27^+^), M1 (CD86^+^ F4/80^+^ CD11b^+^), M2 (CD206^+^ F4/80^+^ CD11b^+^), activated DCs (MHC II^+^ CD11c^+^ CD11b^+^), MDSCs (Gr-1^+^ CD11b^+^), CD4^+^ T cells (CD4^+^), Treg cells (CD25^+^ CD4^+^), and cytotoxic CD8^+^ T cells (GZMB^+^) in the spleens of the mice (*n* = 4 biological replicates). (C) Flow cytometric analysis of CD8^+^ T cells (CD8α^+^) and cytotoxic CD8^+^ T cells (IFNγ^+^) in the spleens of the mice (*n* = 4 biological replicates). (D) Flow cytometric analysis of central memory (CD44^+^ CD62L^+^) and effector memory (CD44^+^ CD62L^−^) CD8^+^ T cells in the spleens of the mice (*n* = 4 biological replicates). (E) Flow cytometric analysis of the indicated immune cells in the tumors of the mice. (F) Flow cytometric analysis of CD8^+^ T cells (CD8α^+^) and cytotoxic CD8^+^ T cells (IFNγ^+^) in the spleens of the mice (*n* = 4 biological replicates). (G) Flow cytometric analysis of central memory (CD44^+^ CD62L^+^) and effector memory (CD44^+^ CD62L^−^) CD8^+^ T cells in the spleens of the mice (*n* = 4 biological replicates). The data are presented as the means ± SEMs and were analyzed with an unpaired two-tailed Student’s t test. *n* = 4 biological replicates. NS, no significant difference; ∗*p* ≤ 0.05, ∗∗*p* ≤ 0.01, and ∗∗∗*p* ≤ 0.001.

To evaluate the potential of oncolytic adv for preventing postoperative tumor recurrence, we investigated its ability to activate antitumor immunity. In a 4T1 mouse model of breast cancer, adv was administered via intratumoral injection (Figure 1A). Flow cytometry analysis of the tumor microenvironment and spleen 2 days after the last treatment revealed that adv did not significantly affect innate immune cells, including NK cells, activated NK cells (CD69^+^ and CD11b^+^ CD27^+^), activated dendritic cells (DCs) (major histocompatibility complex [MHC] II^+^), “M1-like” macrophages (M1) (CD86^+^), “M2-like” macrophages (M2) (CD206^+^), or other immune clusters (Figures 1B and 1E). However, with respect to the adaptive immune response, adv treatment significantly increased the proportions of CD8^+^ T cells and cytotoxic CD8^+^ T cells (interferon [IFN]γ^+^ and granzyme B [GZMB]^+^) (Figures 1C and 1F). Remarkably, adv treatment significantly induced the infiltration of central memory CD8^+^ T cells (CD44^+^ CD62L^+^) into the tumor microenvironment and spleen, as well as effector memory CD8^+^ T cells (CD44^+^ CD62L^−^) in the spleen (Figures 1D and 1G). These results indicate that oncolytic adv induces a systemic antitumor immune response by activating memory T cells, supporting its potential use in controlling postoperative tumor recurrence.

### Design and characterization of an oncolytic adv-loaded supramolecular hydrogel

**Figure 2:**
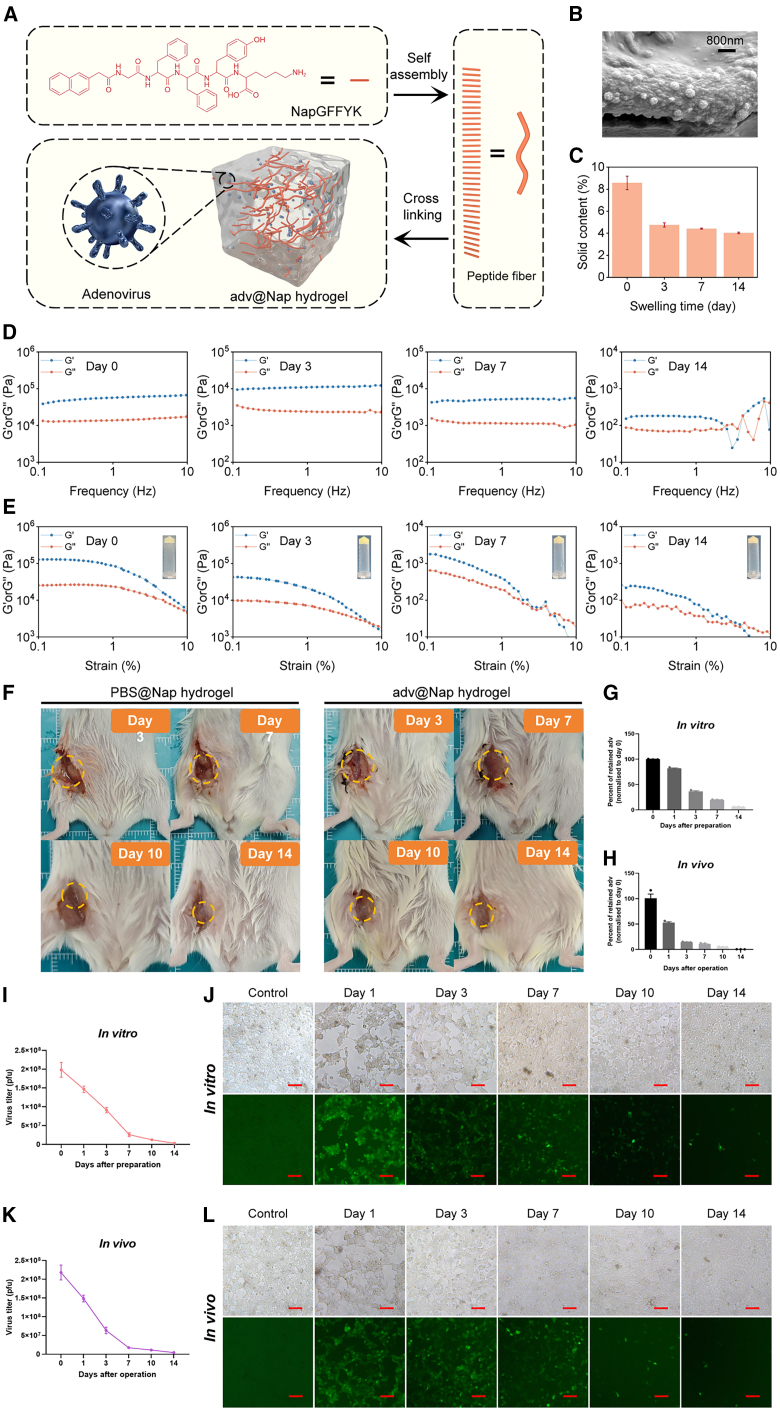
The release of oncolytic adv from hydrogels is a stable and slow process *in vitro* and *in vivo* (A) Design and preparation of the supramolecular hydrogel NapGFFYK gel loaded with oncolytic adv. (B) Representative SEM (scanning electron microscope) image of NapGFFYK gel loaded with adv. Scale bars, 800 nm. (C) Solid content of the adv@Nap gel after several days of immersion *in vitro*. (D and E) Analysis of the rheological properties of the adv@Nap gel on the corresponding days of immersion *in vitro* as a function of frequency (D) and strain (E). The inserts are images of the adv@Nap gel hanging on the bottom of the centrifuge tube without dropping when the tube was flipped upside down for 14 days. (F) Representative images of PBS@Nap gel and adv@Nap gel at the surgical site on day 3, 7, 10, and 14. (G and H) Real-time PCR was used to detect adv hexon in the adv@Nap gel after several days of immersion *in vitro* (G) or *in vivo* (H). (I) TCID_50_ assay for the release rate of adv from the adv@Nap gel after several days of immersion *in vitro.* (J) Representative fluorescence images of adv infection of HEK 293T cells by adv@Nap gel after several days of immersion *in vitro.* Scale bars, 100 μm. (K) TCID_50_ assay for the release rate of adv from the adv@Nap gel after several days of placement *in vivo.* (L) Representative fluorescence images of adv infection of HEK 293T cells by adv@Nap gel after several days of placement *in vivo.* Scale bars, 100 μm. The data are presented as the means ± SEMs. *n* = 3 technical replicates. See also Figures S1 and S2.

The activation of immune memory responses by adv is a prerequisite for its potential to inhibit postoperative tumor recurrence. To fully harness the antitumor immunity-activating capabilities of adv, a strategy of sustained immune activation is needed. Additionally, to address the challenge of OV *in situ* treatment being unfeasible after the removal of the tumor mass, we employed a hydrogel system capable of local drug delivery within the body, which allows for the sustained release of OVs *in situ* as the hydrogel matrix degrades gradually over time. A short peptide, 2-naphthalenyl-glycine-phenylalanine-phenylalanine (NapGFF), was chosen as the motif of the hydrogelator to self-assemble into entangled fibrous network structures of the supramolecular hydrogel. This motif has been widely studied for its value in the construction of peptide hydrogels by our group and others.^53^^,^^54^ Tyrosine and lysine were added to the C terminus to increase self-assembly efficiency in this work, so the intact peptide sequence was NapGFFYK, as schematically depicted in Figure 2A. The π–π stacking and hydrophobic interactions of NapGFFYK led to efficient self-assembly of the peptide to form hydrogels. Adv were dispersed as nanoparticles in the hydrogel supported by peptide fibers (Figure 2B).

The mechanical stability of the adv@Nap gel is important for its application as a viral vector. After 14 days of immersion in simulated body fluid (10% fetal bovine serum [FBS]), the solid content of the adv@Nap gel decreased from 8.5% to 4% and gradually stabilized (Figure 2C), indicating that it would not completely collapse within 14 days *in vivo*. The changes in the rheological properties of the hydrogel with time represent one aspect of its stability. Analysis of the rheological properties revealed that the storage modulus (G′) of the hydrogel was approximately 40∼60 kPa on day 0 and more than 3 times the loss modulus (G”), whereas it decreased to 200 Pa on day 14, approximately 3 times the loss modulus, indicating a solid rather than viscous response of the hydrogel (Figure 2D). Moreover, the hydrogel was mechanically stable up to approximately 6% strain (Figure 2E). The hydrogel could stably hang on the bottom of the centrifuge tube without dropping when the tube was inverted for 14 days, indicating that the hydrogel was physically stable. The same stability test was carried out for the hydrogel without virus (PBS@Nap gel) as for the control group, and the results revealed that the introduction of virus did not affect the mechanical stability of the hydrogel (Figure S1).

In addition, the mechanical stability of the adv@Nap gel and PBS@Nap gel *in vivo* was also studied. On the 3rd, 7th, 10th, and 14th days after implantation of the hydrogels into the mice, visible hydrogel pieces were observed when the surgical suture site was opened (Figure 2F). Analysis of the rheological properties revealed that the storage modulus (4 kPa) and loss modulus (2 kPa) of the hydrogel were similar to those *in vitro*, indicating the continuous mechanical stability of the hydrogel (Figure S2). Notably, we assessed the presence of the viral vector in the hydrogel after several days of immersion in fluid *in vitro* or placement *in vivo*. Real-time PCR was used to assess the hexon adv vector, and the results indicated that the virion was maintained for approximately 14 days and that the number of copies gradually decreased *in vitro* and *in vivo* (Figures 2G and 2H). Furthermore, the rates of adv release from Nap gel *in vitro* (Figures 2I and 2J) and *in vivo* (Figures 2K and 2L) were determined via a 50% tissue culture infectious dose (TCID_50_) assay at different time points, and the infectivity of released adv was tested in HEK293T cells and visualized. Taken together, these results indicate that the supramolecular hydrogel-loaded with oncolytic adv is sufficiently stable and degradable to maintain the extended release and infectivity of the virus both *in vitro* and *in vivo*.

### Placement of adv@Nap gel at the surgical site instantly after tumor resection controls postoperative tumor recurrence

**Figure 3:**
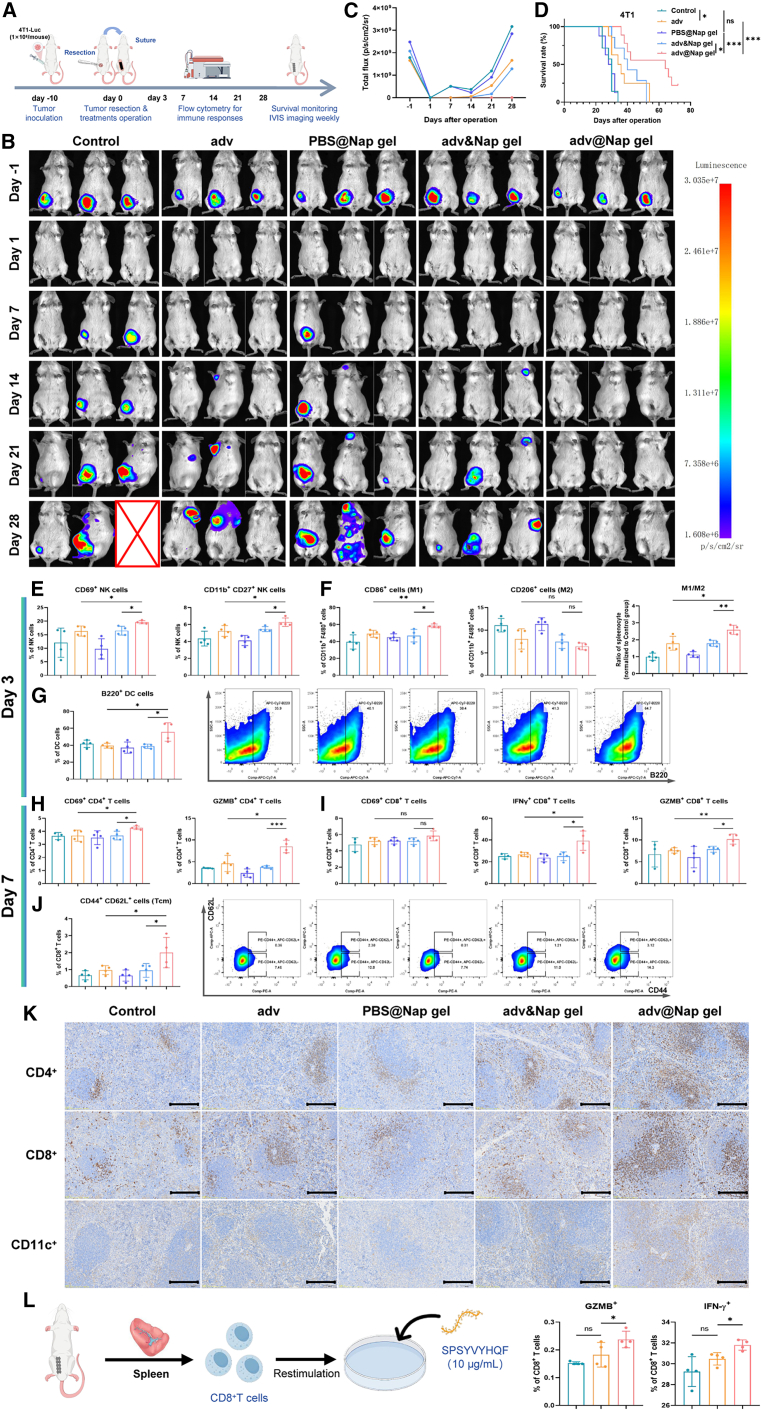
Intraoperative *in situ* treatment with the hydrogel adv@Nap gel controls tumor recurrence and metastasis and activates the antitumor immune response (A) Schematic illustration of the ability of adv@Nap gel to control tumor recurrence in a mouse model of postoperative breast cancer recurrence. (*n* = 7–9 biological replicates). (B) Representative IVIS images of 4T1-Luc cells in all groups at different time points as indicated (*n* = 3 biological replicates). (C) Quantitative statistics of tumor growth in each group according to total fluorescence intensity at the indicated time points (*n* = 3 biological replicates). (D) The postoperative survival curves of the mice in each group are shown, and the significance of differences was analyzed (*n* = 7–9 biological replicates, Kaplan-Meier method with the log rank test). (E‒J) Spleen samples from the mice in each group were analyzed via flow cytometry, and the proportions of various immune cells are shown. (*n* = 4 biological replicates). (K) Representative images of CD4^+^, CD8^+^, and CD11c^+^ immunohistochemical staining of splenocytes from each group 7 days after different treatments. Scale bars, 200 μm. (L) Schematic illustration of the tumor-specific antigen recognition experiment; flow cytometry analysis of cytotoxic CD8^+^ T cells (IFNγ^+^ or GZMB^+^) was performed (*n* = 4 biological replicates). The data are presented as the means ± SEMs and were analyzed with an unpaired two-tailed Student’s t test. NS, no significant difference; ∗*p* ≤ 0.05, ∗∗*p* ≤ 0.01, and ∗∗∗*p* ≤ 0.001. See also Figures S3–S9.

Since the prolonged release of adv from the adv@Nap gel, combined with the induction of adaptive antitumor immune responses, especially cytotoxic effects, as well as central memory CD8^+^ T cells, has been confirmed *in vitro* and *in vivo*, we evaluated the ability of the adv@Nap gel to prevent tumor recurrence following resection in a mouse model of breast cancer. Tumors were resected on day 10 post-inoculation, and adv@Nap gel, PBS@Nap gel, adv solution, or adv mixed with PBS@Nap gel were applied at the surgical site before suturing (Figure 3A)

Tumor recurrence was monitored weekly via an *in vivo* imaging system (IVIS). While no visible tumors remained immediately after surgery, the adv@Nap gel group presented minimal recurrence and metastasis throughout the study, in contrast to significant relapse in other groups (Figure 3B). Quantitative analysis and survival data confirmed that adv@Nap gel treatment most effectively suppressed recurrence and prolonged survival (Figures 3C and 3D).

To determine whether the adv@Nap gel has a direct inhibitory effect on the growth of tumor cells *in situ* after surgery, approximately 5% of the tumor tissue was intentionally retained at the original site during surgical resection, and adv or adv@Nap gel was used for treatment. The residual tumor tissues collected on day 7 were subjected to immunohistochemical staining for detection of tumor cell proliferation (Ki67) and apoptosis (caspase-3) levels. Compared with those in the control group, both the adv and adv@Nap gel treatments significantly decreased intratumoral Ki67 expression but markedly increased caspase-3 levels, indicating that both therapies effectively inhibited tumor cell proliferation and promoted apoptosis (Figures S3A and S3B). Notably, the adv@Nap gel group presented significantly higher Ki67 levels than did the adv group. These results indicate that, while both the adv and adv@Nap gel treatments effectively inhibited tumor cell proliferation, the inhibitory effect of the adv@Nap gel was significantly weaker than that of the adv monotherapy.

Tissue repair and wound healing play critical roles in postoperative recovery following tumor surgery.^55^ Hydrogels are commonly employed clinically as wound dressings to provide an optimal environment for wound healing.^56^ We further evaluated the impact on wound healing. No significant differences in wound closure were observed among the treatment groups (Figure S3C). Histological analysis via Masson’s trichrome staining and CD31 immunohistochemistry confirmed that neither the PBS@Nap nor the adv@Nap gel impaired tissue repair or angiogenesis (Figures S3D‒S3G).

In summary, local application of adv@Nap gel post-resection effectively prevents tumor recurrence without compromising wound healing.

### Preliminary safety assessment of the adv@Nap gel

**Figure 4:**
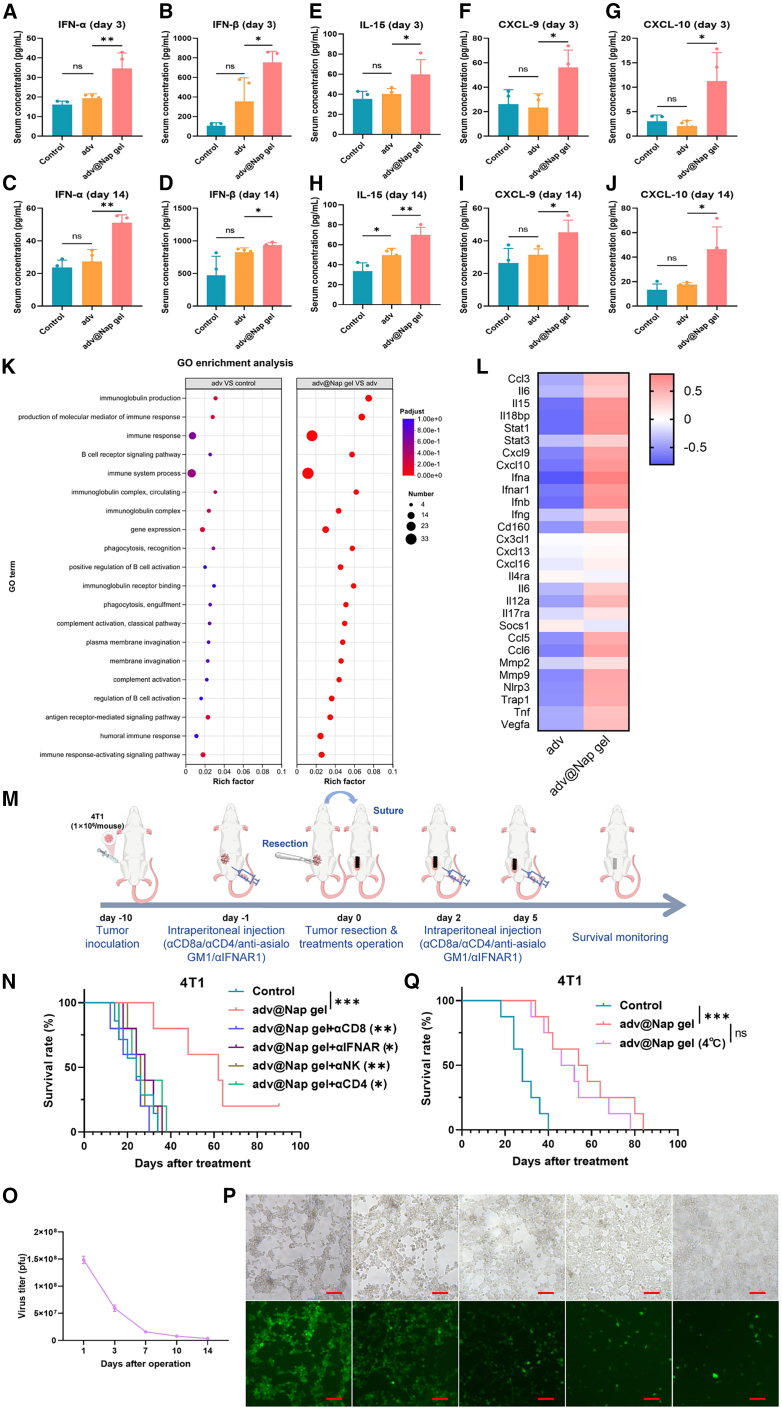
*In situ* placement of the adv@Nap gel induces the type I interferon pathway and chemotaxis (A and B) ELISA of the serum IFN-α (A) and IFN-β (B) concentrations of the mice on day 3. (C and D) ELISA of the serum IFN-α (C) and IFN-β (D) concentrations of the mice on day 14. (E‒G) ELISA results showing the serum IL-15 (E), CXCL-9 (F), and CXCL-10 (G) concentrations of the mice on day 3. (H‒J) ELISA results showing the serum IL-15 (H), CXCL-9 (I), and CXCL-10 (J) concentrations in the mice on day 14. (K) GO analysis of the target gene set between the “adv vs. control” counterpart and the “adv@Nap gel vs. adv” counterpart (*n* = 6 biological replicates). (L) Heatmap of the relative expression levels of the indicated DEGs according to the *Z* score (*n* = 6 biological replicates). (M) Schematic illustration of the mouse antibody deletion experiment (*n* = 5–7 biological replicates). (N) Postoperative survival curves of the mice in each group in the antibody deletion experiment (*n* = 5–7 biological replicates, Kaplan-Meier method with the log rank test). (O) TCID_50_ assay for the release rate of adv from adv@Nap gel preserved at 4°C for 1 week after several days of immersion *in vitro.* (*n* = 3 biological replicates). (P) Representative fluorescence images of adv infection of HEK 293T cells by adv@Nap gel preserved at 4°C for 1 week after several days of immersion *in vitro* (*n* = 3 biological replicates). Scale bars, 100 μm. (Q) Postoperative survival curves of the mice in each group (*n* = 8 biological replicates, Kaplan-Meier method with the log-rank test). The data are presented as the means ± SEMs and were analyzed with an unpaired two-tailed Student’s t test. *n* = 4 biological replicates. NS, no significant difference; ∗*p* ≤ 0.05, ∗∗*p* ≤ 0.01, and ∗∗∗*p* ≤ 0.001. See also Figure S10.

**Figure 5:**
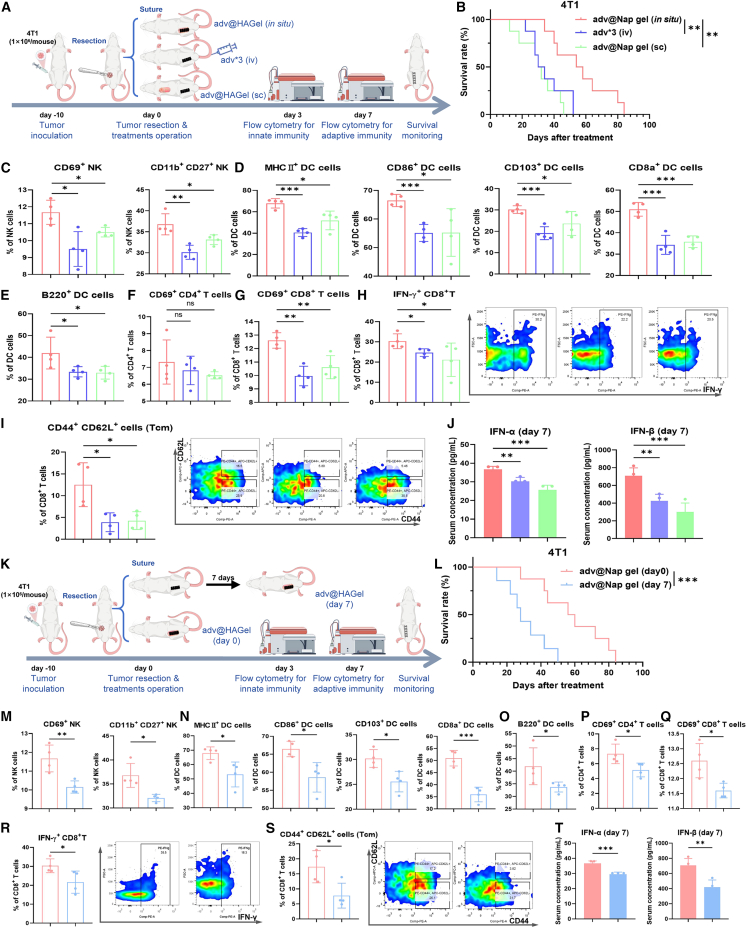
The necessity of instant and *in situ* administration of adv@Nap gel in inducing immune activation to control tumor recurrence after surgery (A) Schematic illustration showing the effects of different dosing modalities for controlling postoperative tumor recurrence (*n* = 8 biological replicates). (B) The postoperative survival curves of the mice in each group (*n* = 8 biological replicates, Kaplan-Meier method with the log rank test). (C‒I) The proportions of various immune cells in the spleen are shown via flow cytometry analysis on day 3 and day 7. (J) ELISA results showing the serum IFN-α and IFN-β concentrations of the mice on day 7. (K) Schematic illustration of the effects of different adv@Nap gel administration times on controlling postoperative tumor recurrence (*n* = 7–8 biological replicates). (L) The postoperative survival curves of the mice in each group (*n* = 7–8 biological replicates, Kaplan-Meier method with the log rank test). (M‒S) The proportions of various immune cells in the spleen are shown via flow cytometry analysis on day 3 and day 7. (T) ELISA of the serum IFN-α and IFN-β concentrations of the mice on day 7. The data are presented as the means ± SEMs and analyzed with an unpaired two-tailed Student’s t test. *n* = 4 biological replicates; ∗*p* ≤ 0.05, ∗∗*p* ≤ 0.01, and ∗∗∗*p* ≤ 0.001.

**Figure 6:**
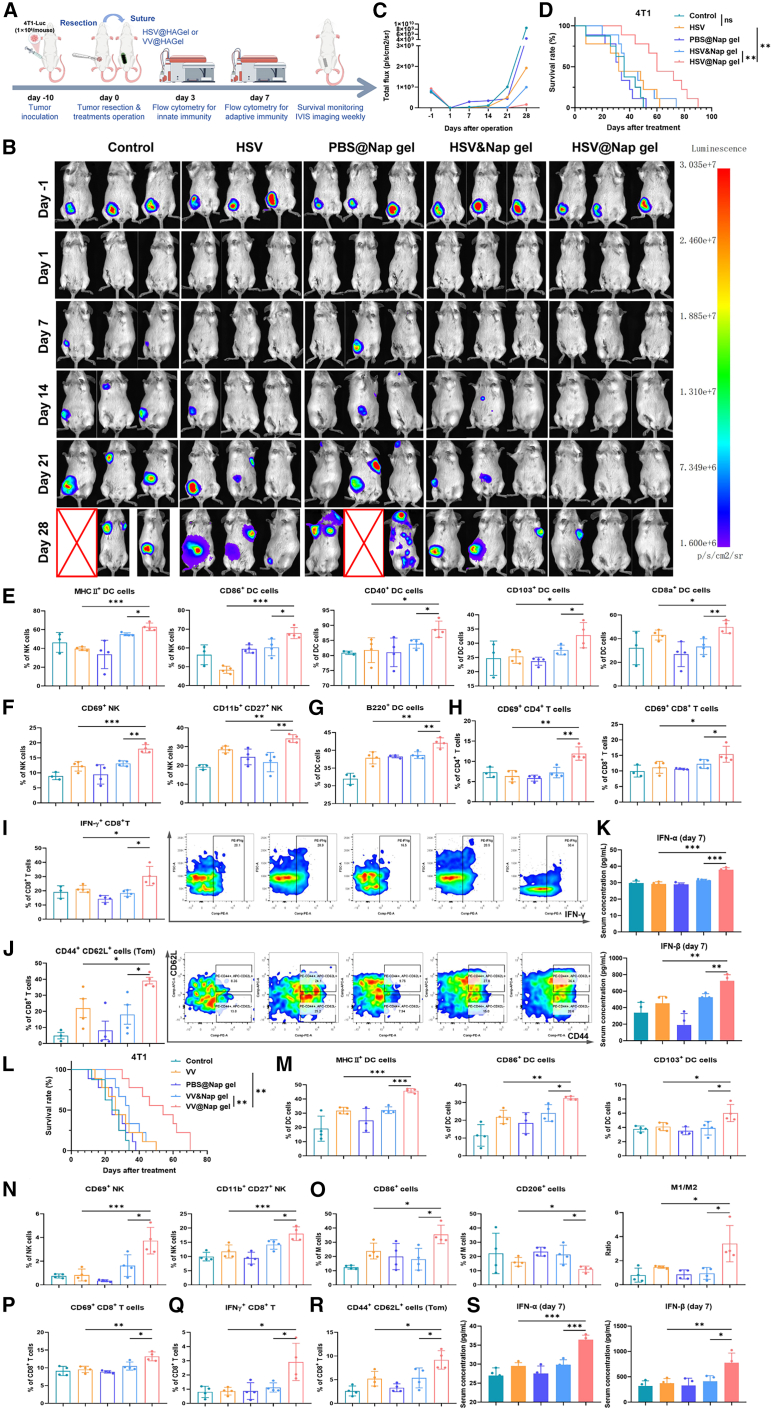
*In situ* treatment with HSV@Nap gel or VV@Nap gel instantly after tumor resection controlled tumor recurrence and metastasis (A) Schematic illustration of the ability of the HSV@Nap gel to control tumor recurrence in a mouse model of postoperative breast cancer recurrence (*n* = 8–9 biological replicates). (B) Representative IVIS images of 4T1-Luc cells in all groups treated with HSV at different time points after surgery (*n* = 3 biological replicates). (C) Quantitative statistics of tumor growth in each group according to total fluorescence intensity at the indicated time points (*n* = 3 biological replicates). (D) Postoperative survival curves of the mice in each group (*n* = 8–9 biological replicates, Kaplan-Meier method with the log rank test). (E‒J) The spleens of the mice in each group were analyzed via flow cytometry, and the proportions of various immune cells are shown. (K) ELISA of the serum IFN-α and IFN-β levels on day 7. (L) Postoperative survival curves of the mice in each group (*n* = 8–9 biological replicates, Kaplan-Meier method with the log rank test). (M‒R) The spleens of the mice in each group were analyzed via flow cytometry, and the proportions of various immune cells are shown. (S) ELISA of the serum IFN-α and IFN-β concentrations on day 7. The data are presented as the means ± SEMs and were analyzed with an unpaired two-tailed Student’s t test. *n* = 4 biological replicates; ∗*p* ≤ 0.05, ∗∗*p* ≤ 0.01, and ∗∗∗*p* ≤ 0.001. See also Figure S11.

**Figure 7:**
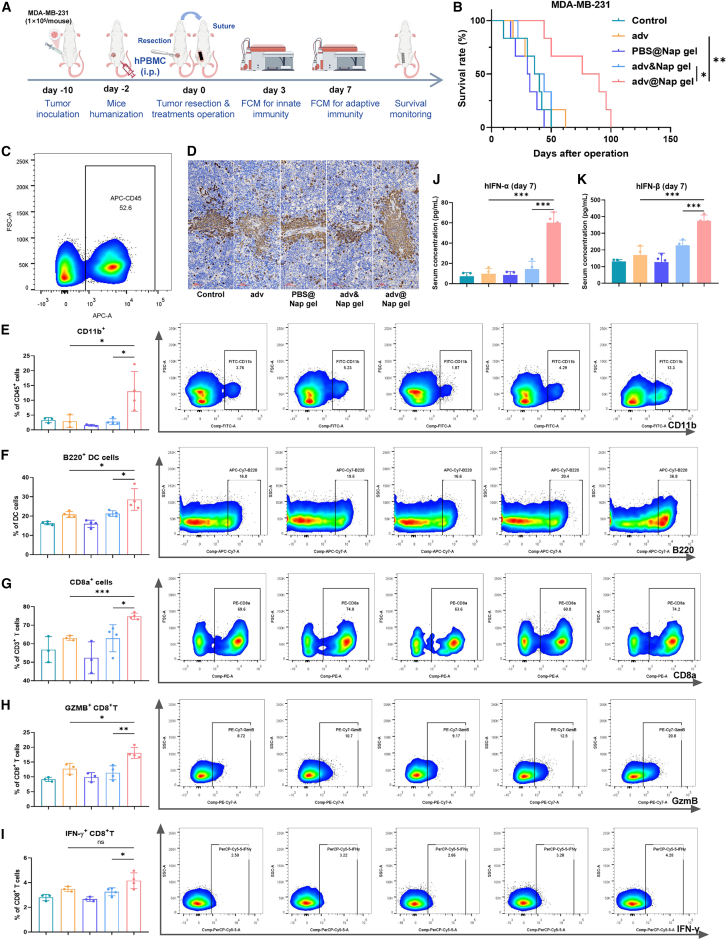
*In situ* treatment with adv@Nap gel instantly after tumor resection controlled tumor recurrence in a mouse model with a humanized immune system (A) Schematic illustration of the ability of the adv@Nap gel to control tumor recurrence in a humanized immune system mouse model of postoperative breast cancer recurrence (*n* = 6 biological replicates). (B) The postoperative survival curves of the mice in each group (*n* = 6 biological replicates, Kaplan-Meier method with the log rank test). (C) Representative flow cytometric analysis of the hCD45^+^ cell cluster on day 3. (D) Representative images of hCD45^+^ immunohistochemical staining of the spleens of the mice in each group 7 days after different treatments. Scale bars, 60 μm. (E‒I) Representative flow cytometric data showing the proportions of the indicated immune cells in the spleens of the mice in each group on day 7 (*n* = 4 biological replicates, unpaired two-tailed Student’s t test). (J and K) ELISA of serum hIFN-α (J) and hIFN-β (K) levels on day 7 (*n* = 4 biological replicates, unpaired two-tailed Student’s t test). The data are presented as the means ± SEMs. NS, no significant difference; ∗*p* ≤ 0.05, ∗∗*p* ≤ 0.01, and ∗∗∗*p* ≤ 0.001. See also Figure S12.

Following the efficacy evaluation, we assessed the safety of the adv@Nap gel *in vivo*. Hematological analysis of mouse blood at 14 days post-surgery revealed that all the parameters were within normal ranges (Figure S4A). Systemic toxicity evaluations, including liver and kidney function (alanine aminotransferase [ALT], aspartate aminotransferase [AST], and blood urea nitrogen [BUN]), revealed no significant differences between the groups (Figure S4B). Histological examination of major organs and surgical sites via H&E staining revealed no obvious toxicity (Figures S4C and S5A). Long-term safety assessed in nonrelapsing mice at 60 days post-surgery also revealed normal hematological, hepatic, and renal parameters (Figure 1, Figure 2, Figure 3, Figure 4, Figure 5, Figure 6, Figure 7S5B and S5C). Body weight remained stable throughout the study (Figure S4D). These results demonstrate that the adv@Nap gel effectively prevents postoperative tumor recurrence without detectable toxicity, supporting its *in vivo* safety and potential for clinical translation.

### Treatment with the adv@Nap gel induces a persistent antigen-specific antitumor immune response

To investigate the mechanism by which the adv@Nap gel inhibits postoperative tumor recurrence and extends survival, immune responses in treated mice were analyzed. Flow cytometry of the splenic samples collected on day 3 revealed significantly greater proportions of activated (CD69^+^) and effector (CD11b^+^ CD27^+^) NK cells, elevated M1 macrophage activation, and an increased M1/M2 ratio in the adv@Nap gel group than in the adv group (Figures 3E and 3F). The proportion of antigen-presenting cells, including MHC II^+^ DCs, CD86^+^ DCs, CD103^+^ DCs, and CD8α^+^ DCs, also increased (Figure S6A), as did the proportion of plasmacytoid DCs (B220^+^), indicating enhanced innate immunity and potential type I interferon pathway activation (Figure 3G). No significant changes in adaptive immunity (activated, cytotoxic, or memory T cells) were detected on day 3 (Figures S6B‒S6D), suggesting that innate immune activation preceded adaptive responses. Therefore, we hypothesized that the activation of innate immunity by adv@Nap gel treatment had just begun on day 3 and had not yet activated the adaptive immune system in time. The immune response levels on day 7 showed that adv@Nap gel significantly increased activated CD4^+^ T cells (CD69^+^) and CD8^+^ T cells (CD69^+^) and central memory T cells (CD44^+^ CD62L^+^), which are critical for long-term antitumor immunity (Figures 3H, 3I, and S6E‒S6H). The immunohistochemical results corroborated these findings (Figure 3K).

To determine the duration of immune activation induced by adv@Nap gel, which releases adv over 14 days, we performed flow cytometry weekly. On day 14, the adv@Nap gel group exhibited significantly stronger innate and adaptive immunity, including antitumor memory responses, than the control groups did (Figures S6I‒S6N). By day 21, innate immunity (NK cells, macrophages, and DCs) returned to baseline, whereas plasmacytoid DCs (B220^+^), adaptive immunity (activated/cytotoxic T cells), and central memory T cells remained elevated (Figures S7A‒S7F). The immune activity decreased to levels comparable to those of the control by day 28 (Figures S7G‒S7J). These results demonstrate that localized extended adv release sustains antitumor immunity for approximately 4 weeks, supporting its efficacy in controlling postoperative recurrence. Moreover, analysis of tumor-draining lymph nodes (tdLNs) and lungs on day 14 revealed immune activation patterns consistent with those of the spleen, demonstrating the broad systemic immunity induced by adv@Nap gel (Figure S8). To determine whether this effect is antigen specific, we isolated CD8^+^ T cells and restimulated them with the 4T1-specific peptide SPSYVYHQF. Compared with the adv group, the adv@Nap group presented significantly greater frequencies of IFNγ^+^ CD8^+^ T cells and GZMB^+^ CD8^+^ T cells, confirming antigen-specific cytotoxicity (Figure 3L). Tumor rechallenge experiments further validated the specificity of the method: mice cured with adv@Nap gel rejected 4T1 rechallenge but developed CT-26 tumors (Figures S9A and S9B). In the 4T1-OVA (ovalbumin) model, OVA-specific CD8^+^ T cells and central memory T cells were significantly expanded and sustained longer in the adv@Nap gel group (Figures S9C and S9D), indicating the induction of durable antigen-specific immune memory.

Taken together, these results suggest that *in situ* loading of adv@Nap gel instantly after tumor resection induces long-term and tumor antigen-specific immune responses by activating innate and adaptive immunity while inducing lasting immune memory and ultimately inhibiting postoperative tumor recurrence and metastasis.

### The placement of adv@Nap gel at the surgical site instantly after tumor resection induces significant systemic type I interferon pathway activation and chemotaxis

Recombinant adenoviral vectors can stimulate plasmacytoid DCs to secrete IFN-α and IFN-β *in vitro* and *in vivo*, inducing the activation of the type I interferon pathway.^57^^,^^58^ The release of adv from the adv@Nap gel stimulated the activation of plasmacytoid DCs (Figure 3G). Therefore, we hypothesized that the control of postoperative tumor recurrence by adv@Nap gel is achieved through the excitation of the type I interferon pathway. Serum analysis confirmed significantly elevated levels of IFN-α and IFN-β in the adv@Nap gel group with those in the adv group on day 3 and 14 after surgery, indicating sustained activation of this pathway (Figures 4A‒4D). In addition, we examined multiple chemokines that recruit immune cells. Interleukin (IL)-15 plays an important role in maintaining the function and development of NK cells and memory CD8^+^ T cells.^59^ Both CXCL-9 and CXCL-10 can bind to CXCR3 to recruit and regulate immune cells, including effector T cells, NK cells, DCs, and macrophages.^60^^,^^61^ ELISAs revealed that treatment with the adv@Nap gel significantly promoted the release of IL-15, CXCL-9, and CXCL-10 on day 3 and 14 compared with that in the adv group (Figures 4E‒4J).

To elucidate the mechanism underlying the inhibition of tumor recurrence by the adv@Nap gel, RNA sequencing (RNA-seq) was performed on spleen samples from the control, adv, and adv@Nap gel groups. Compared with the control group, the adv group presented 192 differentially expressed genes (DEGs), while 278 DEGs were identified between the adv and adv@Nap gel groups (Figure S10A). After 30 overlapping genes were removed, 248 unique DEGs specific to the adv@Nap gel treatment were defined as the target gene set (Figure S10B). Heatmap visualization revealed substantial transcriptomic changes induced by the adv@Nap gel (Figure S10C). Gene Ontology (GO) enrichment analysis revealed that the DEGs between the adv and adv@Nap gel groups were predominantly associated with immune responses, including immunoglobulin production, the generation of molecular mediators of the immune response, and immune system processes. Moreover, compared with the “adv vs. control” counterparts, the “adv@Nap gel vs. adv” DEGs presented more pronounced rich factors (Figure 4K). Further heatmap statistical analysis of specific immune-related cytokines and chemokines revealed that, compared with the adv group, the adv@Nap gel group presented significantly elevated levels of multiple cytokines (including IL-15, Stat1, Ifna, Ifnar1, and Ifnb) and chemokines (Cxcl9, Cxcl10, Ccl5, and Ccl6) (Figure 4L), corroborating the protein-level data. Kyoto Encyclopedia of Genes and Genomes analysis highlighted enrichment in the NOD (nucleotide-binding oligomerization domain)-like receptor, JAK (Janus kinase)-STAT (signal transducer and activator of transcription), and cGMP (cyclic guanosine monophosphate)-PKG (cGMP-dependent protein kinase) signaling pathways (Figure S10D). The JAK-STAT pathway is a canonical downstream cascade of type I interferon signaling^62^^,^^63^ that is activated by elevated Stat1 expression, demonstrating that the adv@Nap gel enhances systemic immunity through sustained type I interferon and JAK-STAT activation to suppress recurrence.

To further verify the necessity of the type I interferon pathway for adv@Nap gel to control tumor recurrence, IFNAR (IFN alpha receptor)-inhibiting antibodies were used to block this pathway. Consequently, the protective effect of the adv@Nap gel against tumor recurrence was diminished (Figure 4M). Moreover, we used blocking antibodies for NK cells, CD4^+^ T cells, and CD8^+^ T cells to confirm that these immune cells are essential for the successful control of tumor recurrence by adv@Nap gel (Figure 4N).

To illustrate the clinical translational potential of the adv@Nap gel, we demonstrated that the adv@Nap gel stored at 4°C for 1 week maintained similar release kinetics and infectivity to freshly prepared adv@Nap gel, along with effective control of postoperative tumor recurrence (Figures 4O‒4Q).

Taken together, these results suggest that *in situ* placement of the adv@Nap gel instantly after tumor resection induces systemic type I interferon pathway activation and chemotaxis and that the type I interferon pathway, as well as its downstream immune cells, is indispensable for inhibiting tumor recurrence.

### Controlling postoperative tumor recurrence with adv@Nap gel requires *in situ* placement instantly after surgery

To confirm the necessity of *in situ* adv@Nap gel placement at the resection site for immune activation and recurrence control, we used two other common dosing modalities: the subcutaneous placement of adv@Nap gel and the intravenous administration of adv (Figure 5A). The survival of the mice that received the *in situ* adv@Nap gel was significantly prolonged (Figure 5B). Flow cytometry revealed markedly greater levels of innate immune cells in this group (Figures 5C–5E), along with significant enrichment of activated CD8^+^ T (CD69^+^) cells, cytotoxic CD8^+^ T (IFNγ^+^) cells, and central memory CD8^+^ T cells (CD44^+^ CD62L^+^), but not activated CD4^+^ T (CD69^+^) cells (Figures 5F‒5I). The serum levels of IFN-α and IFN-β were also the highest in the orthotopic treatment group (Figure 5J). These results underscore that *in situ* placement is essential for optimal activation of type I interferon signaling, innate and adaptive immunity, memory formation, and recurrence suppression.

Notably, we also demonstrated the necessity of instant placement of the adv@Nap gel (Figure 5K). The survival of the mice treated with adv@Nap gel *in situ* on day 7 was significantly shorter than that of the mice treated with adv@Nap gel instantly after surgery (Figure 5L). In terms of immune system activation, as expected, both innate immunity and adaptive immunity, as well as immune memory levels, were significantly lower after *in situ* treatment with adv@Nap gel on day 7 (Figures 5M‒5S). The levels of cytokines in the type I interferon pathway were also decreased (Figure 5T). These results indicate the importance of timely treatment with adv@Nap gel; specifically, timely placement of adv@Nap gel during surgery is essential for its efficacy.

### Oncolytic HSV or oncolytic VV loaded in Nap gels also controls postoperative tumor recurrence

To evaluate whether the Nap gel delivery platform is broadly applicable to other OVs beyond adv, we tested HSV, another widely studied OV in clinical trials,^30^^,^^31^^,^^64^^,^^65^^,^^66^^,^^67^ loaded into the hydrogel (HSV@Nap gel) in the same postoperative breast cancer model (Figure 6A). The IVIS imaging results revealed that *in situ* treatment with HSV@Nap gel instantly after tumor resection effectively suppressed tumor recurrence, although relapses occurred after 4 weeks (Figures 6B and 6C), which was consistent with the survival benefit (Figure 6D). As before, we also measured the ratio of innate and adaptive immune cells on day 3 and day 7. The results were also similar to those previously described (Figures 6E‒6J). Consistently, the concentrations of both IFN-α and IFN-β increased significantly in response to HSV@Nap gel treatment (Figure 6K). These results indicate that *in situ* treatment with HSV@Nap gel instantly after tumor resection can effectively induce an antitumor immune response and control tumor recurrence.

In another validated trial using VV, similar to adv and HSV hydrogels, VV@Nap gel effectively controlled tumor recurrence and metastasis, significantly prolonged survival in mice, and induced the activation of innate and adaptive antitumor immune responses, as well as the type I interferon pathway (Figures 6L‒6S and S11). Specifically, the VV@Nap gel-treated mice exhibited no tumor recurrence, suggesting that, compared with the HSV@Nap gel, the VV@Nap gel is more effective at controlling tumor recurrence and metastasis after surgery.

These results suggest that OVs (other than adv) loaded in Nap gel can achieve *in situ* extended immune activation instantly after tumor resection and effectively control tumor recurrence and metastasis. These results greatly expand the application prospects of OV-loaded Nap gels in antitumor immunotherapy.

### Enhancing the application potential of adv@Nap gel in controlling tumor recurrence

To validate the feasibility of translating previous research findings into clinical practice, it is necessary to verify the ability of the adv@Nap gel to control tumor recurrence in additional tumor models. To this end, a mouse model of melanoma was developed in which B16F10 cells were used (Figure S12A). Consistent with findings in orthotopic breast cancer models, *in situ* adv@Nap gel administration instantly after tumor resection effectively controlled melanoma recurrence (Figure S12B). Similarly, the treatment also activated both innate and adaptive immune responses, ultimately inducing immune memory formation (Figures S12C‒S12H).

Humanized mouse models are essential for validating OV drugs that show promising efficacy in preclinical studies before clinical trials. We established a humanized mouse model using immunodeficient C-NKG mice engrafted with human triple-negative breast cancer MDA-MB-231 cells and human peripheral blood mononuclear cells (Figure 7A). The survival of mice treated *in situ* with adv@Nap gel post-resection was significantly prolonged (Figure 7B). Successful humanization was confirmed by flow cytometry and immunohistochemistry detection of hCD45^+^ cells in the splenic tissue (Figures 7C and 7D). Immune profiling revealed increased proportions of innate immune cells (CD11b^+^ CD45^+^ and B220^+^ plasmacytoid DCs) in the adv@Nap gel group (Figures 7E and 7F). By day 7, adaptive immunity was also enhanced, with higher proportions of CD8^+^ T cells and cytotoxic CD8^+^ T cells (GZMB^+^ or IFNγ^+^) in the experimental groups than in the control groups (Figures 7G‒7I). Consistent with previous findings, the serum levels of IFN-α and IFN-β were significantly elevated (Figures 7J and 7K), confirming the activation of type I interferon signaling in a humanized context.

These results indicate that in a humanized mouse model, *in situ* placement of adv@Nap gel instantly after tumor resection can induce activation of the antitumor immune response and type I interferon pathway and control tumor recurrence after surgery. These findings indicate the potential of the OVs@Nap gel for translation to clinical practice and provide a basis for further clinical translational research.

## Discussion

In the context of cancer immunotherapy, the roles of innate and adaptive immunity are paramount. Innate immunity, the initial response to neoplastic cells, plays a pivotal role in sculpting an antitumor inflammatory milieu within the tumor microenvironment and in augmenting subsequent adaptive immune responses.^68^ Innate immune cells are crucial players in tumor immunotherapy. In this study, we found that *in situ* administration of the adv@Nap gel instantly after tumor resection activated innate immune cells, such as NK cells and DCs, and facilitated coordination between innate and adaptive immune cells, such as cytotoxic T cells. This activation of immune cells serves as a collective indicator of the success of our therapeutic strategy in controlling postoperative tumor recurrence.

Central memory CD8^+^ T (Tcm) cells are vital for the persistence of antitumor immunity and the prevention of tumor recurrence due to their longevity, rapid expansion, and differentiation into effector T cells upon re-exposure to tumor antigens.^69^ Furthermore, the maintenance of Tcm populations is indispensable for long-term immunological memory and surveillance, which is critical for preventing tumor recurrence.^70^ In comparison, effector memory T (Tem) cells do not seem to play a dominant role in controlling tumor recurrence, which may be due to the different roles they fulfill. Tcm cells, which reside primarily in secondary lymphoid organs, mediate long-term immune memory, whereas Tem cells rapidly clear pathogens and circulate in peripheral tissues. Our findings align with this distinction: improved survival in mice was consistently correlated with increased Tcm proportions, suggesting that this metric may predict the ability of immunotherapy to control post-surgical recurrence.

The type I interferon pathway plays a pivotal role in both innate and adaptive immunity.^62^ The activation of this pathway is crucial for the development of immunopathology, particularly in autoimmune inflammatory diseases, where type I IFNs (IFN-α and IFN-β) are implicated.^71^ Type I IFNs elicit antiviral, antiproliferative, and immunomodulatory responses by binding to the type I IFN receptor, which consists of the IFNAR1 and IFNAR2 transmembrane proteins.^72^ Our study revealed that *in situ* extended release of adv activated the type I IFN pathway, which is essential for enhancing both innate and adaptive immunity against tumors. Like prior reports,^73^ we confirmed that type I IFNs, particularly IFN-α and -β from activated DCs,^74^ enable tumor immune recognition by enhancing antigen presentation and effector T cell activation. These results underscore the dual role of the type I IFN pathway in antiviral defense and coordinated anticancer immunity.

OVs can induce systemic antitumor immune responses via multiple mechanisms, including the enhancement of antigen presentation, modulation of the TME, and activation of immune cells.^31^^,^^75^^,^^76^ However, we found that adv alone did not activate innate immunity, as shown in Figure 1. This may be attributed to the low dosage of adv we used. Notably, we found that extending the adv exposure period facilitated significant activation of innate and adaptive immunity. This discovery broadens the potential scope of strategies for increasing the degree of immune response activation with OVs or other immunomodulatory drugs. In addition to increasing the dosage, which may lead to unexpected toxic side effects, the duration of action can also be extended to achieve this goal. Additionally, the type of OV itself is one of the factors to consider when formulating immunotherapy strategies, as OVs have variable properties and different capacities to activate the immune system.

Hydrogels have emerged as versatile platforms in the field of tumor immunotherapy.^77^^,^^78^ The application of hydrogels in tumor immunotherapy is multifaceted. Oklu et al. demonstrated that hydrogels can serve as reservoirs for immunotherapeutic agents, allowing the controlled release of these agents directly at the tumor site.^79^ Moreover, Ribbeck et al. reported that hydrogels can also be used to create an immunoprotective barrier at the tumor site,^80^ which can help prevent the rapid clearance of OVs by the host immune system. We verified that the adv@Nap gel can maintain long-term release, thereby achieving sustained activation of the immune response. Our findings underscore the potential of hydrogel-based delivery systems for increasing the efficacy of OVs and other immunotherapeutic strategies in cancer treatment.

While our hydrogel currently achieves maximum sustained release of OVs over 14 days, feasible strategies to further prolong the release duration remain. The key to sustained release lies in effectively entrapping OVs within the hydrogel matrix. In this study, we employed hydrogen bonding and other weak interactions inherent in supramolecular peptide self-assembly to construct a hydrogel and retain viruses. The incorporation of higher-energy chemical bonds or modifications to the self-assembled architecture of peptides could enhance hydrogel stability, thereby enabling extended viral retention. For example, terminal modification of self-assembling peptides with dopamine,^53^ followed by electrooxidation to dopamine quinone, significantly improves the mechanical robustness of the hydrogel. Furthermore, rational peptide sequence design can induce the formation of supramolecular structures such as α helices or β sheets during self-assembly, thereby reinforcing hydrogel integrity. Previous studies have demonstrated that incorporating phenylalanine into peptide sequences promotes the formation of microscale nanofibers at the supramolecular level,^81^ providing inspiration for the construction of more robust hydrogel frameworks.

Several substantial challenges remain in the clinical translation of our hydrogel-based therapeutic formulation. First, the off-target effects of OVs require genetic engineering modifications to increase the specificity of viral targeting. Additionally, the biocompatibility of hydrogel products, including immune rejection, drug delivery efficiency, and residual cross-linking agents, is particularly prominent. Furthermore, there is a significant technological gap from laboratory research to industrial production, with a fundamental contradiction between the controllability of the production environment and the demand for scale-up. Finally, hydrogel-based therapies also need to address multilevel regulatory barriers, which are reflected in complex application requirements, long approval cycles, and strict technical standards.

Overall, *in situ* extended immune activation by OVs immediately after tumor resection can be utilized to control postoperative tumor recurrence. Our findings clearly demonstrate the significant impact of the immediacy of immunotherapy administration, *in situ* drug delivery, and sustained immune activation on prognosis after tumor surgery, providing a theoretical basis for the clinical application of immunotherapy in managing postoperative recurrence. Additionally, the use of OV-loaded hydrogels represents a vital treatment option for the clinical control of postoperative recurrence.

### Limitations of the study

Although we observed prolonged postoperative survival in a mouse model with this therapeutic approach, tumor recurrence and death ultimately occurred in the majority of the mice. This finding indicates that our treatment method, while effective, is not completely curative. Additionally, the therapeutic efficacy of our OV-loaded hydrogel strategy in scenarios other than postoperative recurrence, including both solid tumors and hematological malignancies, remains to be investigated. The efficacy of an OV-based strategy alone may be limited in the treatment of more complex and highly aggressive tumors. Future research should focus on exploring the potential of combining OV-based strategies with other immunotherapeutic strategies to overcome tumor immune evasion and improve therapeutic outcomes.

## Resource availability

### Lead contact

Further information and requests for resources and reagents should be directed to and will be fulfilled by the lead contact, Junhua Wu (wujunhua@nju.edu.cn).

### Materials availability

The authors declare that all results supporting the findings of this study are available within the paper and its supplemental information.

### Data and code availability

• All the data reported in this paper will be shared by the lead contact upon request. The RNA-seq datasets have been deposited in NCBI with accession number SRA: PRJNA1305683.
• This paper does not report original code.
• Any additional information required to reanalyze the data reported in this work paper is available from the lead contact upon request.

## Acknowledgments

The research was supported by the Key R&D Program of Shandong Province (202502); the Shandong Provincial Natural Science Foundation (ZR2025MS1306); the Shandong Provincial Laboratory Project (SYS202202); the 10.13039/501100001809National Natural Science Foundation of China (82272819 and 81972888); the Research Project of Jinan Microecological Biomedicine Shandong Laboratory (JNL-2025008B, JNL-2025009B, JNL-2025011B, JNL-2025010B, JNL-2025012B, and JNL-2023017D); and the 10.13039/501100013058Primary Research and Development Plan of Jiangsu Province (BE2022840).

## Author contributions

Conceptualization, J.W., B.X., T.G., and C.G.; methodology, C.G., T.G., S.W., R.X., L.K., Y.Z., Q.X., and J.W.; investigation, C.G., T.G., S.W., R.X., L.K., Y.Z., and Q.X.; writing – original draft, C.G. and T.G.; writing – review and editing, C.G., J.W., T.G., and L.K.; funding acquisition, Q.X., C.J., X.G., and J.W.; supervision, C.G., B.X., C.J., X.G., and J.W.

## Declaration of interests

The authors declare no competing interests.

## STAR★Methods

### Key resources table

**Table:** 

REAGENT or RESOURCE	SOURCE	IDENTIFIER
**Antibodies**		

APC anti-mouse CD45.1 Antibody	BioLegend	Cat#110714; RRID: AB_313503
FITC anti-human CD45 Antibody	BioLegend	Cat#103108; RRID: AB_312973
APC/Cyanine7 anti-mouse CD3 Antibody	BioLegend	Cat#100221; RRID: AB_2242784
FITC anti-mouse NKp46 Antibody	Invitrogen	Cat#A14752; RRID: AB_2534268
PE anti-mouse CD49b Antibody	BioLegend	Cat#103506; RRID: AB_313029
FITC anti-mouse CD4 Antibody	BioLegend	Cat#100406; RRID: AB_312691
PE/Cyanine7 anti-mouse CD4 Antibody	BioLegend	Cat#100422; RRID: AB_312707
PerCP/Cyanine5.5 anti-mouse CD8α Antibody	BioLegend	Cat#100734; RRID: AB_2075238
PerCP/Cyanine5.5 anti-mouse CD103 Antibody	BioLegend	Cat#121416; RRID: AB_312790
PE/Cyanine7 anti-mouse CD11c Antibody	BioLegend	Cat#117318; RRID: AB_493568
PE anti-human/mouse GZMB Antibody	BioLegend	Cat#372207; RRID: AB_2687031
PE/Cyanine7 anti-mouse IFNγ Antibody	BioLegend	Cat#505825; RRID: AB_2295770
APC/Cyanine7 anti-mouse CD25 Antibody	BioLegend	Cat#101917; RRID: AB_2650982
PE/Cyanine7 anti-mouse CD69 Antibody	BioLegend	Cat#104512; RRID: AB_493564
APC/Cyanine7 anti-mouse/rat/human CD27 Antibody	BioLegend	Cat#124225;RRID: AB_2565791
PE/Cyanine7 anti-mouse F4/80 Antibody	BioLegend	Cat#123114; RRID: AB_893478
PE anti-mouse CD86 Antibody	BioLegend	Cat#159203; RRID: AB_2832567
PerCP/Cyanine5.5 anti-mouse CD206 Antibody	BioLegend	Cat#141716; RRID: AB_2561992
APC/Cyanine7 anti-mouse I-A/I-E Antibody	BioLegend	Cat#107627; RRID: AB_2069377
APC/Cyanine7 anti-mouse/human CD45R/B220 Antibody	BioLegend	Cat#103224; RRID: AB_313007
PE anti-mouse Ly-6G/Ly-6C (Gr-1) Antibody	BioLegend	Cat#108407; RRID: AB_313372
APC anti-mouse CD62L Antibody	BioLegend	Cat#104411; RRID: AB_313098
PE anti-mouse/human CD44 Recombinant Antibody	BioLegend	Cat#163609; RRID: AB_2924492
PE/Cyanine7 anti-mouse H-2K^b^ bound to SIINFEKL Antibody	BioLegend	Cat#141607; RRID: AB_11219193
APC anti-human CD45 Antibody	BioLegend	Cat#304037; RRID: AB_2562049
FITC anti-human CD3 Antibody	BioLegend	Cat#317306; RRID: AB_571906
PE anti-human CD8α Antibody	BioLegend	Cat#300908; RRID: AB_314111
APC anti-mouse/human CD11b Antibody	BioLegend	Cat#101212; RRID: AB_312795
PE/Cyanine7 anti-human CD11c	BioLegend	Cat#980606; RRID: AB_2894600
PerCP/Cyanine5.5 anti-human IFNγ Antibody	BioLegend	Cat#506527; RRID: AB_2566186
*InVivo* MAb anti-mouse IFNAR-1	BioXCell	Cat#BE0241; RRID:AB_2687723
*InVivo* MAb anti-mouse CD8α	BioXCell	Cat#BE0061; RRID:AB_1125541
*InVivo* MAb anti-mouse CD4	BioXCell	Cat#BE0003-1; RRID:AB_1107636
Anti asialo GM1 (Rabbit)	FUJIFILM Wako	Cat#986-10001; RRID: AB_516844
Anti-CD4 Rabbit pAb	Servicebio	Cat#GB11064; RRID: AB_2904187
Anti-CD8 alpha Mouse mAb	Servicebio	Cat#GB12068; RRID: AB_2905512
Anti-CD11c Mouse mAb	Servicebio	Cat#GB12059; RRID: AB_3716399
Anti-human CD45 Rabbit pAb	Servicebio	Cat#GB115428; RRID: AB_3106955
Anti-Caspase-3 Rabbit pAb	Servicebio	Cat#GB11009-1; RRID: AB_3661664
Anti-CD31 Rabbit pAb	Servicebio	Cat#GB113151; RRID: AB_2923131
Anti-Ki67 Rabbit pAb	Servicebio	Cat#GB111141; RRID: AB_3096315

**Bacterial and virus strains**		

adv	Provided by Professor Jiwu Wei	N/A
HSV	Wuhan Binhui	N/A
VV	Wuhan Binhui	Cat#VR-1540-ATC

**Biological samples**		

hPBMC	Blood from the healthy donor	N/A

**Chemicals, peptides, and recombinant proteins**		

Dulbecco’s modified Eagle’s medium (DMEM)	Invitrogen	Cat#10564011
Fetal Bovine Serum (FBS)	Invitrogen	Cat#A4766801
Streptomycin/penicillin	Invitrogen	Cat#15140122
293 Pro	BasalMedia	Cat#F431166
Puromycin	MCE	Cat#HY-B1743A
Permeabilization kit	eBioscience	Cat#00-5523-00
Collagenase Type IV	gibco	Cat#17104019
D-Luciferin	aladdin	Cat#L120798
isoflurane	RWD	Cat#R510-22-10
DMSO	aladdin	Cat#D103276
DAPI	Beyotime	Cat#P0131
Peptide Nap-GFFYK	Bankpeptide	N/A
Xylene	Sinopharm	Cat#10023418
n-Butyl alcohol	Sinopharm	Cat#100052190
Hydrochloric acid	Sinopharm	Cat#10011028
Universal tissue fixative (neutral)	Servicebio	Cat#G1101
Masson dye solution set	Servicebio	Cat#G1006
Haematoxylin Differentiate Solution	Servicebio	Cat#G1039
Neutral gum	SCRC	Cat#10004160
Antigen peptide SPSYVYHQF	GenScript	N/A

**Critical commercial assays**		

HiScript II One Step RT-PCR Kit	Vanzyme	Cat#P611-01
Mouse IFN-α ELISA kit	Enzyme-linked	Cat#ml002017
Mouse IFN-β ELISA kit	Enzyme-linked	Cat#ml001982
Mouse IL-15 ELISA kit	Enzyme-linked	Cat#ml002279
Mouse CXCL9 ELISA kit	Enzyme-linked	Cat#ml037904
Mouse CXCL10 ELISA kit	Enzyme-linked	Cat#ml063284
Mouse CD8^+^ T cell Isolation Kit	Selleck	Cat#B90011
DAB chromogenic agent for histochemical kit	Servicebio	Cat#G1212

**Deposited data**		

RNA-seq analysis of mice spleen samples	NCBI SRA	SRA: PRJNA1305683

**Experimental models: Cell lines**		

4T1 cells	ATCC	Cat#CRL-2537
4T1-Luc cells	Ubigene	Cat#YC-B004-Luc-P
4T1-OVA	Provided by Professor Jiwu Wei	N/A
MDA-MB-231 cells	ATCC	Cat#CRM-HTB-26
MDA-MB-231-Luc cells	Ubigene	Cat#YC-D005-Luc-P
B16F10	ATCC	Cat# CRL-6475
CT-26 cells	ATCC	Cat#CRL-2638
HEK 293T cells	ATCC	Cat#CRL-1573

**Experimental models: Organisms/strains**		

Mouse: BALB/c	GemPharmatech	SN#000651
Mouse: C57BL/6J	GemPharmatech	SN#000664
Mouse: C-NKG	Cyagen	SN#C001316

**Software and algorithms**		

AniView X	Biolight	N/A
GraphPad Prism	GraphPad	N/A
FlowJo 10	Tree Star	N/A
Adobe Illustrator	Adobe	N/A

### Experimental model and study participant details

#### Cell lines

The mouse breast cancer cell lines 4T1, 4T1-luciferase and 4T1-OVA; the mouse melanoma cell line B16F10; the human breast cancer cell line MDA-MB-231; and the mouse colorectal cancer cell line CT-26 were cultured in DMEM supplemented with 10% FBS, 100 U/mL penicillin, and 0.1 mg/mL streptomycin. For 4T1-luciferase and 4T1-OVA, 0.5 μg/mL puromycin was added to the culture medium. All the cells were incubated at 37°C with 5% CO_2_.

#### Mice

All animal experiments were carried out in accordance with the guidelines approved by the Ethics Committee of The Affiliated Drum Tower Hospital, Medical School of Nanjing University. Six-to eight-week-old mice were used in these experiments. Female wild-type BALB/c mice were purchased from GemPharmatech Co., Ltd. (Nanjing, China). Female NOD-Prkdc-scid IL2rgem1/Cyagen (C-NKG) mice were purchased from Jiangsu Cyagen Biosciences Co., Ltd. (Shanghai, China). The mice were housed under specific pathogen-free (SPF) conditions at a temperature of 18°C–24°C with water and food and maintained on 12 h light/dark cycles.

### Method details

#### Preparation of the NapGFFYK hydrogel

A dimethyl sulfoxide (DMSO) solution of NapGFFYK (80 mg/mL) and a virus solution (2 × 10^9^ pfu/mL for adv (human type V adenovirus), 1 × 10^8^ pfu/mL for HSV (HSV-1, strain F), or 1 × 10^7^ pfu/mL for VV (strain Elstree)) of the same volume were rapidly mixed. The mixture was shaken on a vortex mixer (Scientific Instruments, USA) for 20 s, and a translucent hydrogel was formed instantaneously.

#### Solid content of the adv@Nap hydrogel

The hydrogels were weighed, and the wet weight was recorded as W_1_. Then, the hydrogel samples were lyophilized. The dry weight was recorded again as W_2_. The solid content was calculated as W_2_/W_1_×100%.

#### Rheological measurement and scanning electron microscopy of the adv@Nap hydrogel

The hydrogels were carefully transferred to a rheometer plate (Thermo Scientific Haake RheoStress 6000) with a spatula prior to measurement. The rheology experiments were then carried out in strain-sweep mode with a strain amplitude range of 0.1%–10% at 1 Hz and in frequency-sweep mode with a frequency range of 0.1–10 Hz at 1% strain (gap: 1.5 mm; temperature: 20°C). Scanning electron microscopy (SEM) images were captured via a Gemini500 scanning electron microscope set at an acceleration voltage of 10 kV.

#### qRT‒PCR

To detect the viral copy number in cells infected with adv via qRT‒PCR, HEK 293T cells were collected at different time points. Each sample was treated with 500 μL of proteinase K lysis buffer (containing 100 μg/mL proteinase K, 50 mmol/L potassium chloride, 10 mmol/L Tris, and 0.5% Tween). The cells were lysed by repeated pipetting. The lysates were incubated at 56°C for 45 min to completely lyse the HEK 293T cells and release the viral genomic DNA. The proteinase K was then inactivated by heating the lysates at 100°C for 10 min. The prepared viral genomic DNA templates were used for subsequent qPCR amplification. The primers used for amplification were as follows: hexon forward primer (5′-TGGGCATCCTACACCAACAC) and reverse primer (5′-AGTGCGCCCATGGACATAAA).

#### TCID_50_ assay

To detect the rate of adv release from Nap gel, the remaining hydrogel pieces were collected after several days of immersion *in vitro* or placement *in vivo*, and the virus titer was determined via the TCID_50_ method. Briefly, HEK 293T cells were seeded into 96-well plates (1×10^4^) and infected with serially diluted samples. Ninety-six hours later, wells with green fluorescent cells under a fluorescence microscope were defined as positive. The adenovirus titers were measured via a TCID_50_ assay as follows: 0.7 × 10×10ˆ (1 + S (D-0.5)), where S = log10 (dilution) and D = the sum of the GFP-positive ratio of each dilution.

#### Establishment of the tumor model and treatment

For the orthotopic breast tumor model, 4T1 cells (1×10^6^/mouse) were injected into the fourth mammary fat pads of female BALB/C mice. When the tumor volume reached approximately 80–100 mm^3^, the mice were randomly divided into a control group and an adv group, and 100 μL of PBS or 2 × 10^9^ cfu/mL adv solution was intratumorally injected into the mice on days 0, 2, and 4. Two days after the last adv administration, the spleens and tumors of the mice were harvested, and single-cell suspensions were prepared, stained and analyzed via flow cytometry.

For the orthotopic postoperative recurrent breast tumor model, 4T1, 4T1-luciferase or 4T1-OVA (for tetramer staining) cells (1×10^6^/mouse) were injected into the fourth mammary fat pads of female BALB/C mice. On the 10th day after tumor inoculation, the mice were continuously anesthetized with isoflurane, and the tumor mass was surgically resected. Then, adv@Nap gel or PBS@Nap gel was placed at the original tumor site, and the incision was sutured. The control group mice were sutured directly after tumor resection. The mice in the adv group were injected with the same amount of adv solution at the tumor site. To demonstrate that adv needs to be loaded into the hydrogel to exert its effects, we compared the adv&Nap gel group, in which the mice were treated with PBS@Nap gel and injected with adv solution at the resected tumor site. After all these operations, each wound was sutured, and the mice were returned to their cages after regaining consciousness.

For the tumor rechallenge experiments, 5×10^5^ 4T1 or 1×10^5^ CT-26 cells were injected into the underarm or contralateral underarm of each mouse, respectively. The weights of the mice were monitored. The tumor length (L) and width (W) were measured every two days via a digital caliper, and the tumor size (V) was calculated as V = (L×W^2^)/2. The mice were sacrificed if the tumor volume exceeded 1500 mm^3^.

For the subcutaneous melanoma model, female C57BL/6J mice were inoculated with B16F10 cells (5×10^5^/mouse) at the right underarm. On the 7th day after inoculation, when the tumor had grown to approximately 100 mm^3^, the tumor was surgically removed (day 0), and the corresponding operations were performed on each group as previously mentioned. At the indicated time points after the operation, the spleens of the mice were collected, and single-cell suspensions were prepared, stained and analyzed via flow cytometry.

For the humanized breast tumor recurrence model, MDA-MB-231 cells (5×10^5^/mouse) were injected into the fourth mammary fat pads of female C-NKG mice. Eight days later, human peripheral blood (obtained from 30-year-old male volunteers with informed consent) mononuclear cells (hPBMCs) were intraperitoneally injected (5×10^6^/mouse). Two days after tumor inoculation, the mice were randomly divided into different groups and treated as described above.

#### *In vivo* bioluminescence and imaging

The mice were inspected with an IVIS imaging system one day before surgery and weekly after tumor resection for local tumor recurrence and metastasis. Each time before imaging, D-luciferin potassium salt (150 mg/kg) was intraperitoneally injected into the mice, which were then anesthetized with 2% isoflurane. Then, bioluminescence images were captured with an IVIS Spectrum *in vivo* imaging system (AniView X), and unified scales, as well as quantitative statistical results, were provided with the software (AniView X).

#### Biochemistry test for blood composition

To test the safety of the therapeutic measurements, whole blood was collected from the mice 7 days after surgery. For ALT, AST and BUN, serum samples were collected by placing the blood sample at room temperature for 2 h and centrifuging for 15 min at 3000 rpm/min, after which the supernatant was collected for instant detection. For other compositions of the blood, plasma samples were stabilized with EDTA or heparin as an anticoagulant. An automatic biochemical instrument (Chemray 800, Rayto) was subsequently used to detect the composition of the blood, and the results were exported after the automatic biochemical analyzer was tested.

#### Deletion of immune cells and neutralization of IFNAR1

To confirm the requirement of specific immune subsets and type I IFN for the therapeutic effect, antibodies against NK cells (anti-asialo GM1, FUJIFILM Wako, Japan), CD4^+^ T cells (anti-mouse CD4, Bioxcell, West Lebanon, NH, USA), CD8^+^ T cells (anti-mouse CD8α, Bioxcell, West Lebanon, NH, USA), or IFNAR1 (anti-mouse IFNAR-1, Bioxcell, West Lebanon, NH, USA) were intraperitoneally used every 3 days beginning 1 day before surgery. All the antibodies were used at 200 μg/mouse in a timely manner.

#### Flow cytometry

The samples were detected on a BD FACSAria III and analyzed with FlowJo 10. All the antibodies were purchased from BioLegend or eBioscience. For immune cells in the tumor microenvironment, spleen, tdLN or lung tissues were collected and digested with collagenase IV (50 mg/mL) for 2 h at 37°C and filtered with 70 μm sieve mesh to generate single-cell suspensions. The acquired single-cell suspensions were stained with different antibodies. 4′,6-Diamidino-2-phenylindole (DAPI) was used to exclude dead cells before analysis. Fluorescent antibodies, including APC-CD45, FITC-CD45, APC/Cyanine7-CD3, FITC-NKp46, PE-CD49b, FITC-CD4, PE/Cyanine7-CD4, PerCP/Cy5.5-CD8α, APC-CD11b, PE-CD11b, PE/Cyanine7-CD11c, PE-GZMB, PE/Cyanine7-IFNγ, APC/Cyanine7-CD25, PE-Gr-1, PE/Cyanine7-CD69, PerCP/Cy5.5-CD103, APC/Cyanine7-CD27, APC/Cyanine7-B220, PE/Cyanine7-F4/80, PE-CD86, PerCP/Cy5.5-CD206, APC/Cyanine7-MHC II, PE-Gr1, APC-CD62L, PE-CD44, and PE/Cyanine7-H-2K^b^-SIINFEKL, were used.

The cells were grouped as follows: lymphocytes (FSC-H, SSC-H), single cells (FSC-H, FSC-A), CD4^+^ T cells (CD4^+^ gated CD3^+^ cells), CD8^+^ T cells (CD8α^+^ gated CD3^+^ cells), activated CD4^+^ T cells (CD69^+^ gated CD4^+^ cells), activated CD8^+^ T cells (CD69^+^ gated CD8α^+^ cells), cytotoxic CD8^+^ T cells (IFNγ^+^ or GZMB^+^ gated CD8α^+^ cells), Treg cells (CD25^+^ gated CD4^+^ T cells), NK cells (NKp46^+^ or CD49b^+^ gated CD45^+^ cells), activated NK cells (CD69^+^ or CD11b^+^ CD27^+^ gated NKp46^+^ or CD49b^+^ cells), DCs (CD11c^+^ MHC II^+^ gated CD11b^+^ CD45^+^ cells), activated DCs (CD8α^+^, CD103^+^ or CD86^+^ gated DCs), plasmacytoid DCs (B220^+^ gated DCs), macrophages (CD11b^+^ F4/80^+^ gated CD45^+^ cells), “M1-like” macrophages (CD86^+^ gated macrophages), “M2-like” macrophages (CD206^+^ gated macrophages), central memory T cells (CD44^+^ CD62L^+^ gated CD8α^+^ or CD4^+^ T cells), effector memory T cells (CD44^+^ CD62L^−^ gated CD8α^+^ or CD4^+^ T cells), tumor antigen-specific Tcm by tetramer staining (CD44^+^ CD62L^+^ gated H-2K^b^-SIINFEKL^+^ CD8^+^ T cells).

To verify tumor-specific antigen recognition, the antigen peptide SPSYVYHQF (GenScript) was used at a concentration of 10 μg/mL. Splenocytes were extracted (Mouse CD8^+^ T cell sorting kit, Selleck) from the mice in the corresponding groups and cultured for 6 h with the antigen peptide. Cytotoxic CD8^+^ T cells (IFNγ^+^ or GZMB^+^ gated CD8α^+^ cells) were analyzed via flow cytometry.

#### RNA sequencing (RNA-seq)

Spleen samples were taken from the mice on Day 14 after surgery. Total RNA was extracted from the tissue via TRIzol (Vazyme) Reagent according to the manufacturer’s instructions. RNA purification, reverse transcription, library construction and sequencing were performed at Shanghai.

Majorbio Biopharm Biotechnology Co., Ltd. (Shanghai, China) according to the manufacturer’s instructions. The RNA-seq transcriptome library was prepared following Illumina Stranded mRNA Prep, Ligation (San Diego, CA) using 1 μg of total RNA. After quantification with a Qubit 4.0, the sequencing library was generated on a NovaSeq X Plus platform (PE150) via a NovaSeq Reagent Kit. These sequence read archive (SRA) data are available at the NCBI (SRA: PRJNA1305683).

#### Enzyme-linked immunosorbent assay (ELISA)

To test cytokine levels in the mice, whole blood was collected from the eyes of the mice. After being incubated at room temperature for 2 h, the serum was extracted via centrifugation for 15 min at 3000 rpm/min, after which the supernatant was collected. The expression levels of the cytokines IFNα, IFNβ, IL-15, CXCL-9 and CXCL-10 were analyzed with ELISA kits (Shanghai Enzyme-linked Biotechnology Co., Ltd.) according to the manufacturer’s protocols.

#### Immunohistochemistry (IHC)

Mouse tissues were fixed in 4% neutral buffered formalin, paraffin embedded, cut into 5 μm sections and subjected to H&E and histochemical immune staining. First, the paraffin sections were dewaxed with water: the sections were gradually dewaxed with environmentally friendly solution I for 10 min, environmentally friendly dewaxing solution II for 10 min, environmentally friendly dewaxing solution III for 10 min, anhydrous ethanol I for 5 min, anhydrous ethanol II for 5 min, anhydrous ethanol III for 5 min, and distilled water. For H&E staining, the sections were treated with hematoxylin solution for 1–2 min or eosin solution for 0.5 min. Then, the samples were washed with water for 2 min, dehydrated with 95% alcohol (2× changes), treated with absolute alcohol (2× changes) and cleared in xylene (3× changes) for 3 min each. Finally, the samples were covered with a coverslip. For IHC, dewaxed sections were subjected to antigen retrieval (repair fluid and repair conditions were determined according to the tissue). Then, endogenous peroxidase activity was blocked, and the cells were incubated with serum. After that, the sections were incubated with primary antibodies. Next, the secondary antibody was added, and the samples were color developed with 3,3′-diaminobenzidine (DAB). The positive color was brown and yellow. After that, the nuclei were stained with hematoxylin. Finally, the slices were removed from xylene to dry slightly, and the slices were sealed with glue.

### Quantification and statistical analysis

The survival of the mice was analyzed via the Kaplan‒Meier method with the log-rank test. All the data are presented as the means ± SEMs. Two-tailed Student’s t tests were used for two-group comparisons, and ordinary two-way ANOVA was used for multiple group comparisons. NS, no significant difference; ∗*p* ≤ 0.05, ∗∗*p* ≤ 0.01, ∗∗∗*p* ≤ 0.001.

## Supplemental information

Document S1. Figures S1–S12

Document S2. Article plus supplemental information
