# Supplementary material for: In situ extended immune activation instantly after tumor resection by oncolytic virus controls postoperative tumor recurrence
Source: Cell Rep Med. 2025 Oct 3;6(10):102399. doi: 10.1016/j.xcrm.2025.102399 (PMC12629831; doi:10.1016/j.xcrm.2025.102399)
Supplement: Document S1. Figures S1–S12 [file mmc1.pdf]

Cell Reports Medicine, Volume *6*

Supplemental information

In situ extended immune activation instantly after tumor resection by oncolytic virus controls postoperative tumor recurrence

Ciliang Guo, Tian Gao, Bin Xue, Louqian Zhang, Shuo Wang, Rongrong Xiao, Lingkai Kong, Yuxin Zhang, Qilei Xin, Yi Cao, Xiaosong Gu, Chunping Jiang, and Junhua Wu

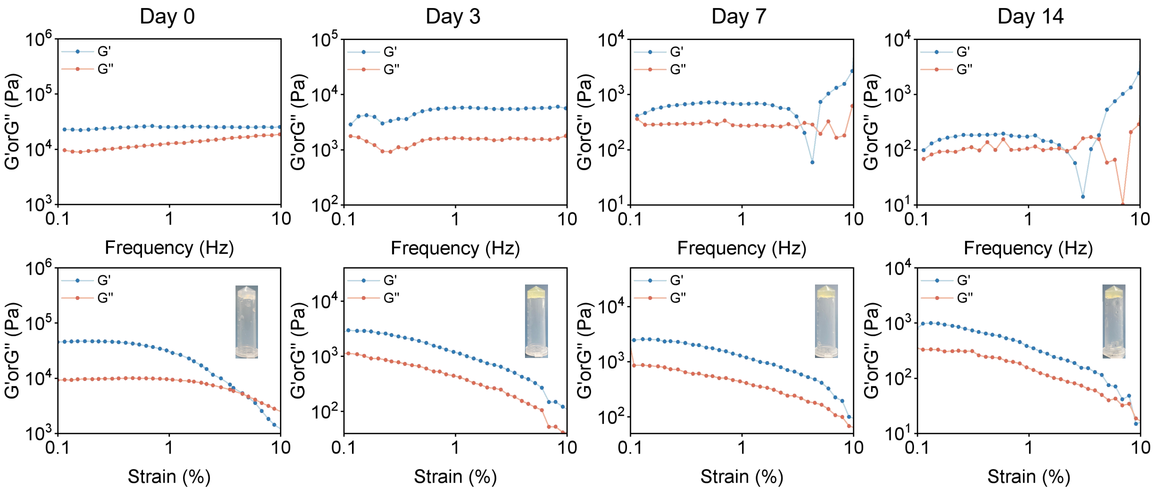

Figure S1. Mechanical stability test of the PBS@Nap gel *in vitro*. Related to Figure 2. Analysis of the rheological properties of the PBS@Nap gel on the corresponding days of immersion *in vitro* as a function of frequency and strain. The inserts are images of the PBS@Nap gel hanging on the bottom of the centrifuge tube without dropping when the tube was flipped upside down for 14 days (n = 3 technical replicates).

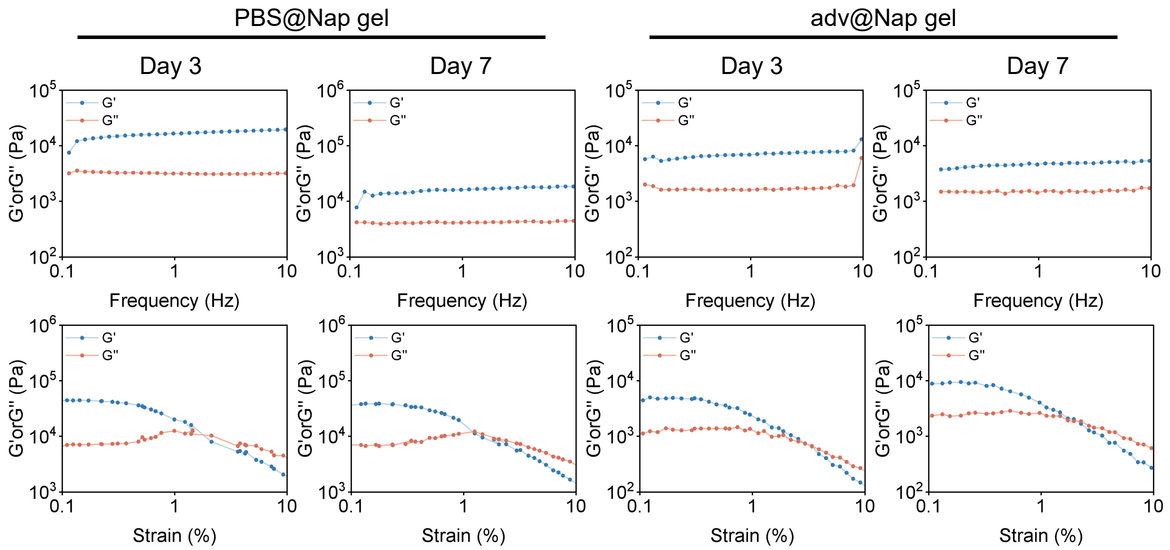

Figure S2. Mechanical stability test of the PBS@Nap gel and adv@Nap gel *in vivo*. Related to Figure 2.

Analysis of the rheological properties of the PBS@Nap gel and adv@Nap gel on the corresponding days of placement *in vivo* as a function of frequency and strain.

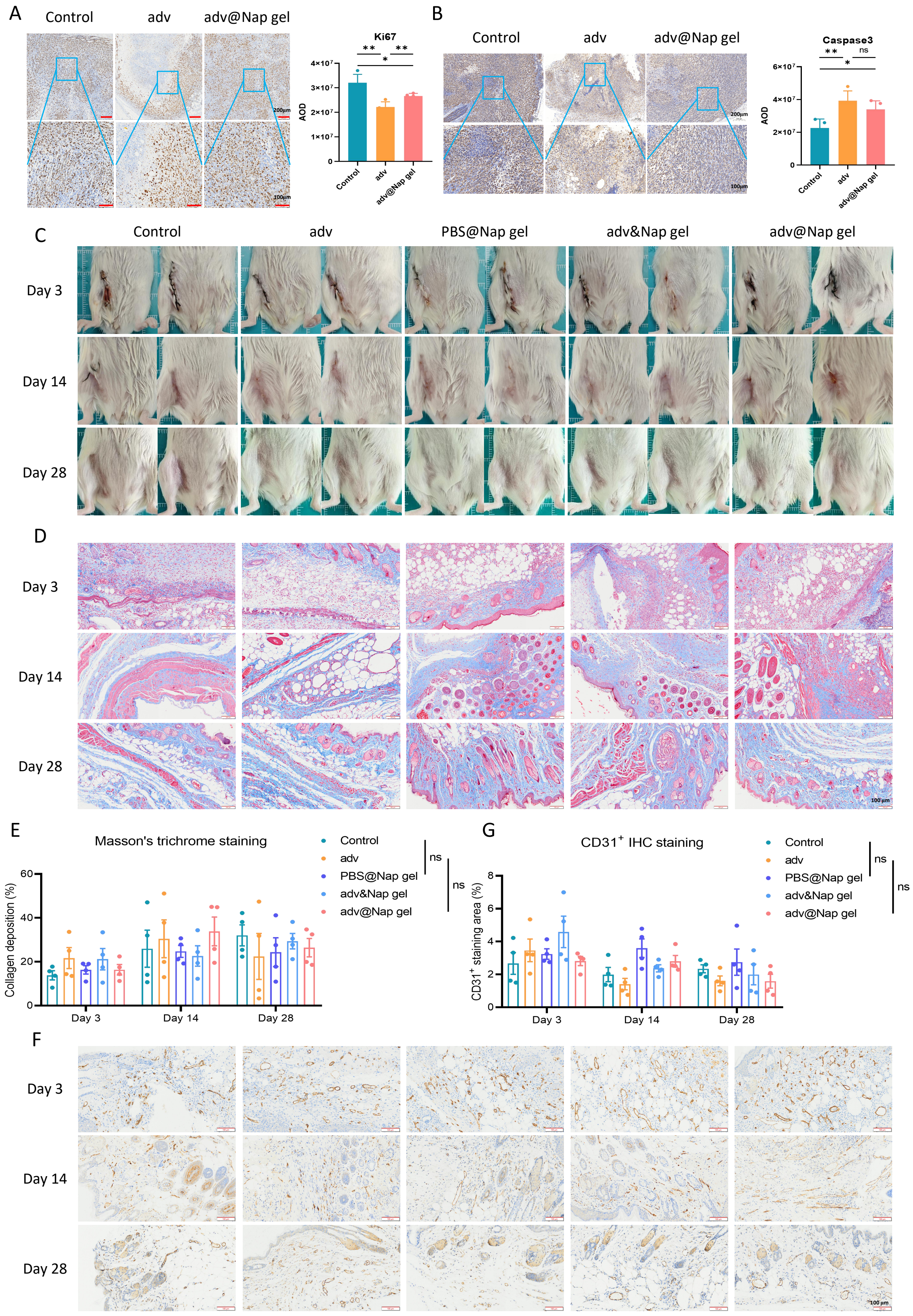

A
Control
adv
adv@Nap gel
200μm
100μm
Ki67
AOD
Control
adv
adv@Nap gel
B
Caspase3
C
Control
adv
PBS@Nap gel
adv&Nap gel
adv@Nap gel
Day 3
Day 14
Day 28
D
100 μm
E
Masson's trichrome staining
Collagen deposition (%)
ns
G
CD31⁺ IHC staining
CD31⁺ staining area (%)
F

Figure S3. Determination of the effects of intraoperative *in situ* treatment with the hydrogel adv@Nap gel on residual tumor tissue and the hydrogel system on wound healing. Related to Figure 3.

(A) Representative images and quantification of Ki67 immunohistochemical staining of residual tumor tissue at the surgical site on Day 7 after surgery. Scale bars, upper: 200 μm, bottom: 100 μm.

(B) Representative images and quantification of Caspase3 immunohistochemical staining of residual tumor tissue at the surgical site on Day 7 after surgery. Scale bars, upper: 200 μm, bottom: 100 μm.

(C) Representative photographs showing the wound tissue healing process in mice on day 3, 14 and 28 after surgery.

(D) Representative images of Masson's trichrome-stained wound tissue from each group on day 3, 14 and 28 after surgery. Scale bars, 100 μm.

(E) Quantification of collagen deposition density in different groups on day 3, 14 and 28 after surgery.

(F) Representative images of $CD31^+$ immunohistochemical staining of the wound tissue from each group on day 3, 14 and 28 after surgery. Scale bars, 100 μm.

(G) Quantification of the $CD31^+$ staining area in different groups on day 3, 14 and 28 after surgery.

The data are presented as the means ± SEMs and were analyzed via ordinary two-way ANOVA. $n = 4$ biological replicates. NS, no significant difference; $*P \leq 0.05$, $**P \leq 0.01$, $***P \leq 0.001$.

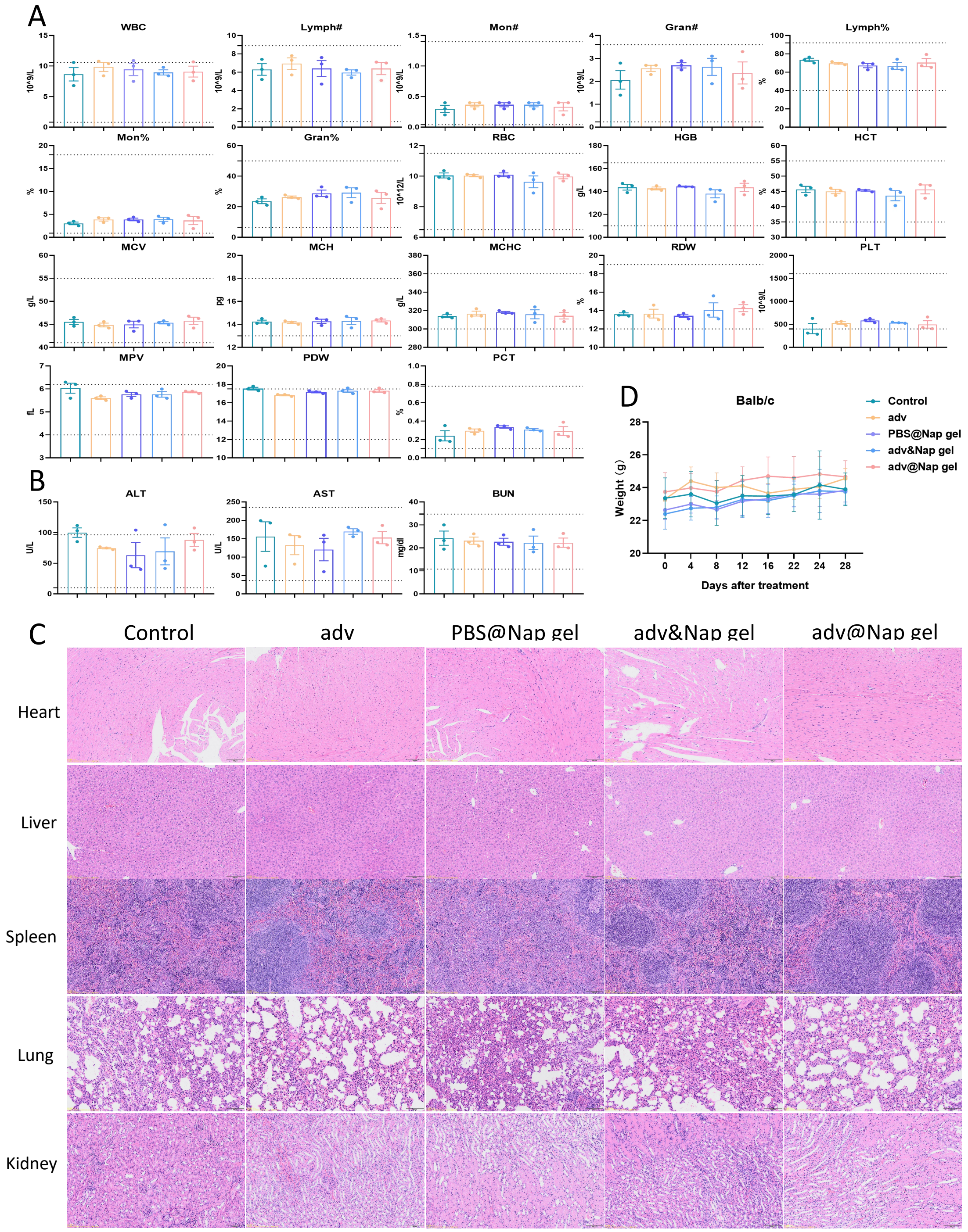

Figure S4. In situ treatment with adv@Nap gel instantly after tumor resection is safe without overt toxic effects. Related to Figure 3.

(A) Composition of whole blood from mice on day 7 after treatment with the adv@Nap gel. WBC, white blood cell; Lymph, lymphocyte; Mon, monocyte; Gran, neutrophil; RBC, red blood cell; HGB, hemoglobin concentration; HCT, hematocrit; MCV, mean corpuscular volume; MCH, mean corpuscular hemoglobin; MCHC, mean corpuscular hemoglobin concentration; RDW, red cell distribution width; PLT, platelet count; MPV, mean platelet volume;

PDW, platelet distribution width; PCT, plateletcrit. The dashed lines indicate established normal ranges (*n* = 3 biological replicates).

(B) The concentrations of alanine aminotransferase (ALT), aspartate aminotransferase (AST), and blood urea nitrogen (BUN) in the serum of the mice on day 7 after treatment with the adv@Nap gel. The dashed lines indicate established normal ranges (*n* = 3 biological replicates).

(C) Representative hematoxylin and eosin (H&E) staining images of major organs, including the heart, liver, spleen, lung and kidney, of mice on day 7 after treatment with adv@Nap gel (*n* = 3 biological replicates). Scale bars, 100 μm.

(D) Body weights of the mice after surgery (*n* = 5 biological replicates).

The data are presented as the means ± SEMs.

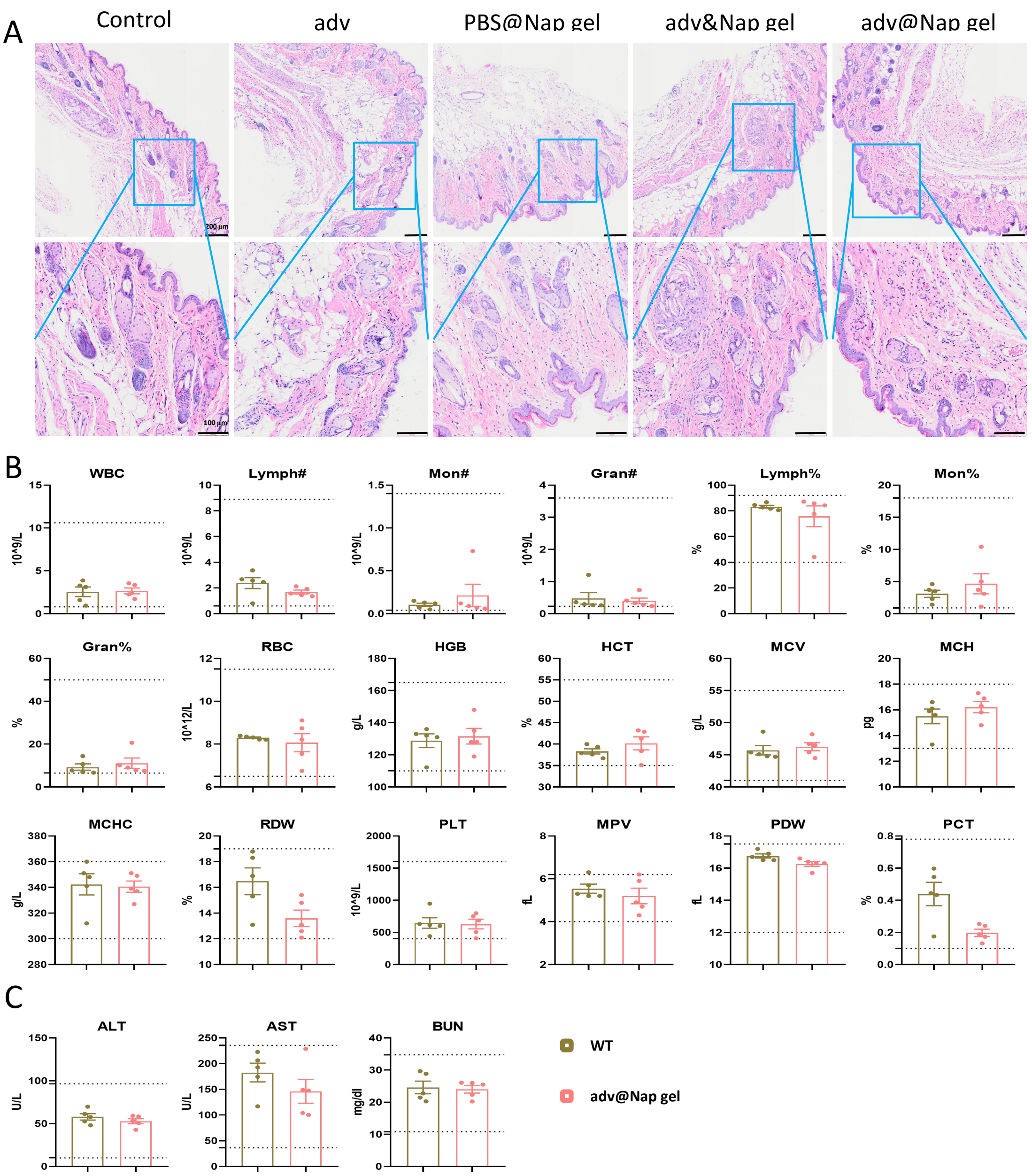

Figure S5. No long-term toxic effects were shown by *in situ* treatment with adv@Nap gel instantly after tumor resection. Related to Figure 3.

(A) Representative H&E staining images of *in situ* surgical tissue on day 21 after treatment with adv@Nap gel (n = 3 biological replicates). Scale bars, upper: 200 μm, bottom: 100 μm.

(B) Composition of whole blood from wild-type (WT) and cured mice after adv@Nap gel treatment.

(C) The concentrations of ALT, AST and BUN in the serum of WT and cured mice after adv@Nap gel treatment. The data are presented as the means ± SEMs. n = 5 biological replicates. The dashed lines indicate established normal ranges.

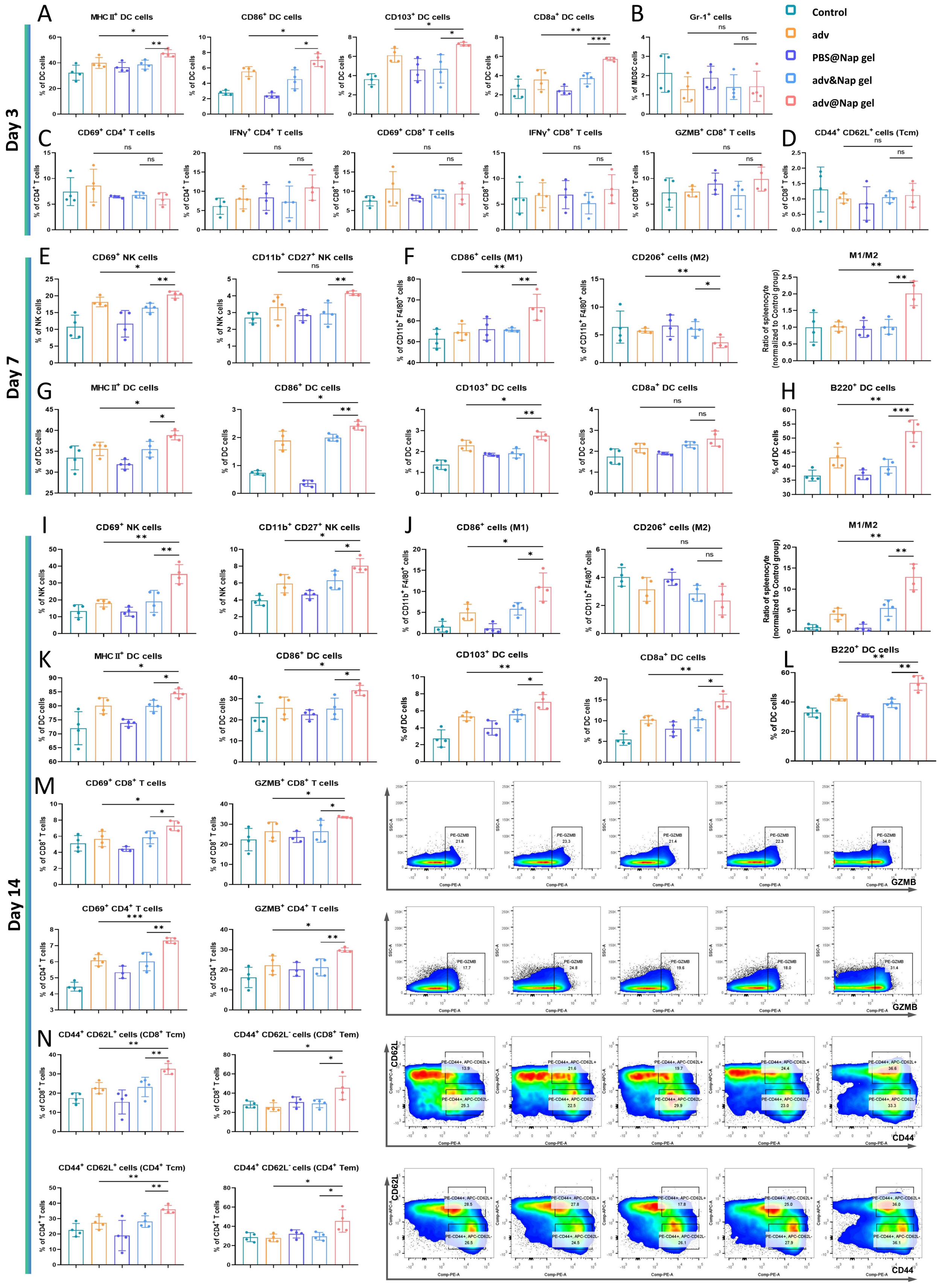

Day 3
Day 7
Day 14
Control
adv
PBS@Nap gel
adv&Nap gel
adv@Nap gel
A
MHC II+ DC cells
CD86+ DC cells
CD103+ DC cells
CD8a+ DC cells
% of DC cells
B
Gr-1+ cells
% of MDSC cells
C
CD69+ CD4+ T cells
IFNγ+ CD4+ T cells
CD69+ CD8+ T cells
IFNγ+ CD8+ T cells
GZMB+ CD8+ T cells
% of CD4+ T cells
% of CD8+ T cells
D
CD44+ CD62L+ cells (Tcm)
E
CD69+ NK cells
CD11b+ CD27+ NK cells
% of NK cells
F
CD86+ cells (M1)
CD206+ cells (M2)
% of CD11b+ F4/80+ cells
M1/M2
Ratio of splenocyte (normalized to Control group)
G
MHC II+ DC cells
CD86+ DC cells
CD103+ DC cells
CD8a+ DC cells
H
B220+ DC cells
I
CD69+ NK cells
CD11b+ CD27+ NK cells
J
CD86+ cells (M1)
CD206+ cells (M2)
M1/M2
K
MHC II+ DC cells
CD86+ DC cells
CD103+ DC cells
CD8a+ DC cells
L
B220+ DC cells
M
CD69+ CD8+ T cells
GZMB+ CD8+ T cells
CD69+ CD4+ T cells
GZMB+ CD4+ T cells
GZMB
SSC-A
Comp-PE-A
PE-GZMB
N
CD44+ CD62L+ cells (CD8+ Tcm)
CD44+ CD62L- cells (CD8+ Tem)
CD44+ CD62L+ cells (CD4+ Tcm)
CD44+ CD62L- cells (CD4+ Tem)
CD62L
CD44
Comp-APC-A
PE-CD44+, APC-CD62L+
PE-CD44+, APC-CD62L-
ns

Figure S6. Intraoperative *in situ* treatment with the hydrogel adv@Nap gel induced a continuous antitumor immune response on day 3, 7 and 14. Related to Figure 3.

Spleen samples from the mice in each group were analyzed via flow cytometry at different time points, and the proportions of various immune cells are shown.

(A) Activated DCs ($MHCII^+$ $CD11c^+$ $CD11b^+$ or $CD86^+$ $CD11c^+$ $CD11b^+$) and DCs that cross-present antigens and generate an antitumor immune response ($CD103^+$ $CD11c^+$ $CD11b^+$ or $CD8\alpha^+$ $CD11c^+$ $CD11b^+$) on Day 3.

(B) MDSCs ($Gr\text{-}1^+$) on Day 3.

(C) Activated $CD4^+$ T cells ($CD69^+$), cytotoxic $CD4^+$ T cells ($IFN\gamma^+$), activated $CD8^+$ T cells ($CD69^+$), and cytotoxic $CD8^+$ T cells ($IFN\gamma^+$ or $GZMB^+$) on Day 3.

(D) Central memory $CD8^+$ T cells ($CD44^+$ $CD62L^+$) on Day 3.

(E) Activated NK cells ($CD69^+$) and high effector NK cells ($CD11b^+$ $CD27^+$) on Day 7.

(F) M1 ($CD86^+$ $F4/80^+$ $CD11b^+$), M2 ($CD206^+$ $F4/80^+$ $CD11b^+$) and the M1/M2 ratio on Day 7.

(G) Activated DCs ($MHCII^+$ $CD11c^+$ $CD11b^+$ or $CD86^+$ $CD11c^+$ $CD11b^+$) and DCs that cross-present antigens and generate an antitumor immune response ($CD103^+$ $CD11c^+$ $CD11b^+$ or $CD8\alpha^+$ $CD11c^+$ $CD11b^+$) on Day 7.

(H) Plasmacytoid DCs ($B220^+$ $CD11c^+$ $CD11b^+$) on Day 7.

(I) Activated NK cells ($CD69^+$) and high effector NK cells ($CD11b^+$ $CD27^+$) on Day 14.

(J) M1 ($CD86^+$ $F4/80^+$ $CD11b^+$), M2 ($CD206^+$ $F4/80^+$ $CD11b^+$) and the M1/M2 ratio on Day 14.

(K) Activated DCs ($MHCII^+$ $CD11c^+$ $CD11b^+$ or $CD86^+$ $CD11c^+$ $CD11b^+$) and DCs that cross-present antigens and generate an antitumor immune response ($CD103^+$ $CD11c^+$ $CD11b^+$ or $CD8\alpha^+$ $CD11c^+$ $CD11b^+$) on Day 14.

(L) Plasmacytoid DCs ($B220^+$ $CD11c^+$ $CD11b^+$) on Day 14.

(M) Activated $CD8^+$ T cells ($CD69^+$), cytotoxic $CD8^+$ T cells ($GZMB^+$) and representative flow cytometry plots, activated $CD4^+$ T cells ($CD69^+$), and cytotoxic $CD4^+$ T cells ($GZMB^+$) and representative flow cytometry plots on Day 14.

(N) Central memory $CD8^+$ T cells ($CD44^+$ $CD62L^+$), effector memory $CD8^+$ T cells ($CD44^+$ $CD62L^-$) and representative flow cytometry plots, central memory $CD4^+$ T cells ($CD44^+$ $CD62L^+$), effector memory $CD4^+$ T cells ($CD44^+$ $CD62L^-$) and representative flow cytometry plots on Day 14.

The data are presented as the means ± SEMs and were analyzed with an unpaired two-tailed Student's t test. $n = 4$ biological replicates. NS, no significant difference; $*P \leq 0.05$, $**P \leq 0.01$, $***P \leq 0.001$.

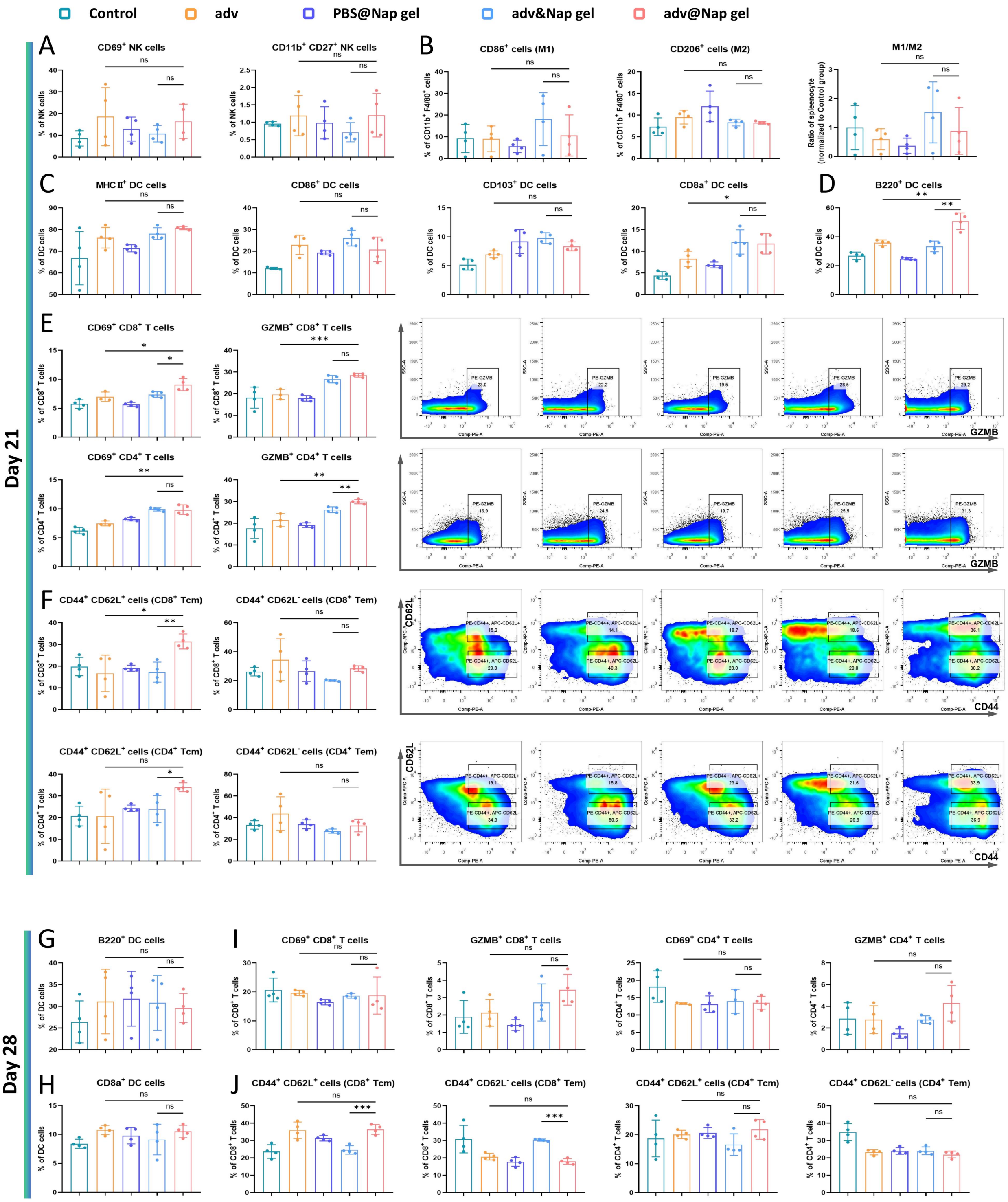

Figure S7. Intraoperative *in situ* treatment with the hydrogel adv@Nap gel induced a continuous antitumor immune response on day 21 and 28. Related to Figure 3.

Spleen samples from the mice in each group were analyzed via flow cytometry at different time points, and the proportions of various immune cells are shown.

(A) Activated NK cells ($CD69^{+}$) and high effector NK cells ($CD11b^{+}$ $CD27^{+}$) on Day 21.

(B) M1 macrophages ($CD86^+$ $F4/80^+$ $CD11b^+$), M2 macrophages ($CD206^+$ $F4/80^+$ $CD11b^+$) and the M1/M2 ratio on Day 21.

(C) Activated DCs ($MHCII^+$ $CD11c^+$ $CD11b^+$ or $CD86^+$ $CD11c^+$ $CD11b^+$) and DCs that cross-present antigens and generate an antitumor immune response ($CD103^+$ $CD11c^+$ $CD11b^+$ or $CD8\alpha^+$ $CD11c^+$ $CD11b^+$) on Day 21.

(D) Plasmacytoid DCs ($B220^+$ $CD11c^+$ $CD11b^+$) on Day 21.

(E) Activated $CD8^+$ T cells ($CD69^+$), cytotoxic $CD8^+$ T cells ($GZMB^+$) and representative flow cytometry plots, activated $CD4^+$ T cells ($CD69^+$), and cytotoxic $CD4^+$ T cells ($GZMB^+$) and representative flow cytometry plots on Day 21.

(F) Central memory $CD8^+$ T cells ($CD44^+$ $CD62L^+$), effector memory $CD8^+$ T cells ($CD44^+$ $CD62L^-$) and representative flow cytometry plots; central memory $CD4^+$ T cells ($CD44^+$ $CD62L^+$), effector memory $CD4^+$ T cells ($CD44^+$ $CD62L^-$) and representative flow cytometry plots on Day 21.

(G) Plasmacytoid DCs ($B220^+$ $CD11c^+$ $CD11b^+$) on Day 28.

(H) DCs that cross-present antigens and generate an antitumor immune response ($CD8\alpha^+$ $CD11c^+$ $CD11b^+$) on Day 28.

(I) Activated $CD8^+$ T cells ($CD69^+$), cytotoxic $CD8^+$ T cells ($GZMB^+$), activated $CD4^+$ T cells ($CD69^+$), and cytotoxic $CD4^+$ T cells ($GZMB^+$) on Day 28.

(J) Central memory $CD8^+$ T cells ($CD44^+$ $CD62L^+$), effector memory $CD8^+$ T cells ($CD44^+$ $CD62L^-$), central memory $CD4^+$ T cells ($CD44^+$ $CD62L^+$), and effector memory $CD4^+$ T cells ($CD44^+$ $CD62L^-$) on Day 28.

The data are presented as the means ± SEMs and were analyzed with an unpaired two-tailed Student's t test. n = 4 biological replicates. NS, no significant difference; $*P \leq 0.05$, $**P \leq 0.01$, $***P \leq 0.001$.

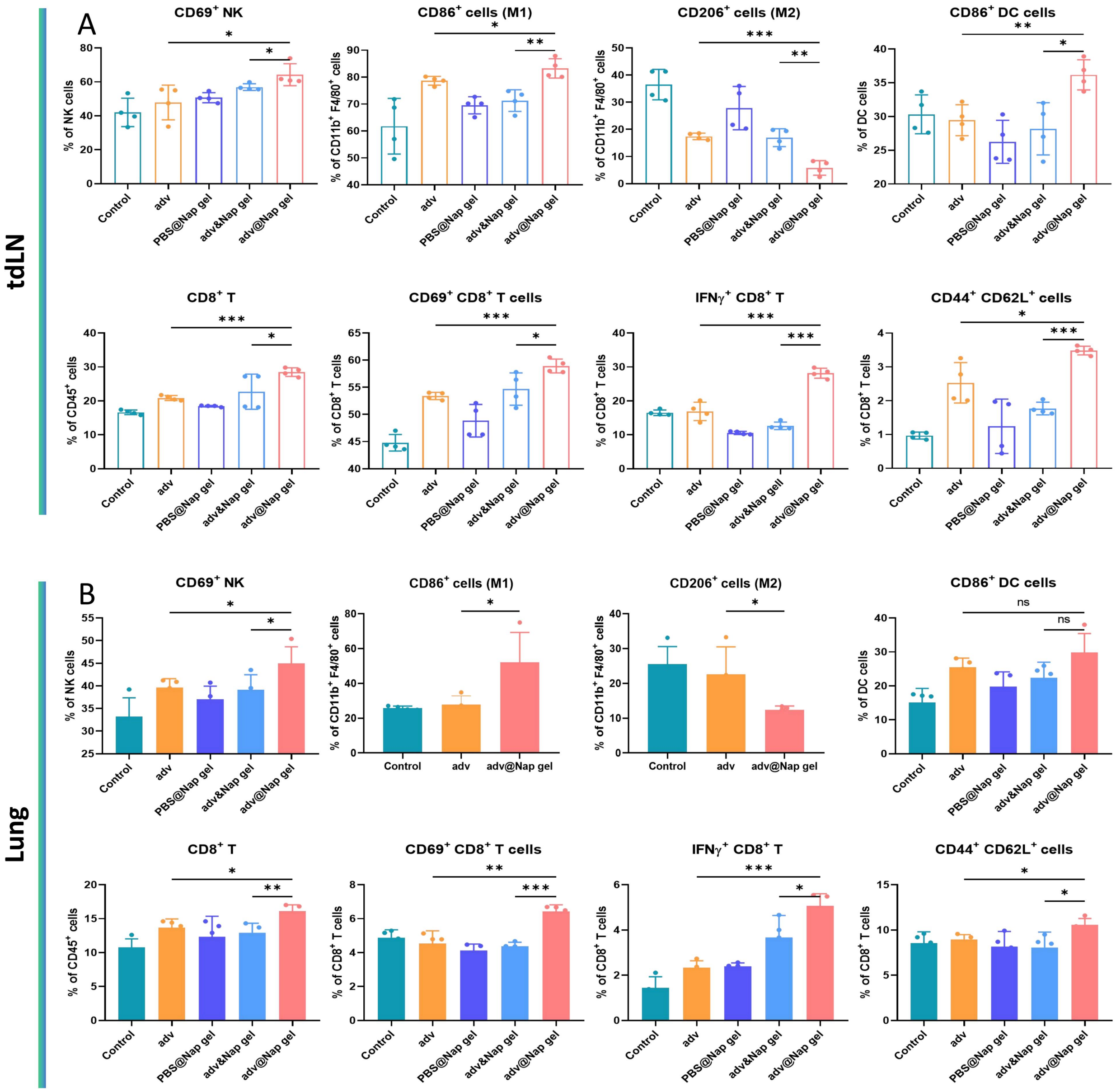

Figure S8. Intraoperative *in situ* treatment with the hydrogel adv@Nap gel induces immune response activation in the tdLN and lungs of mice. Related to Figure 3.

The tdLN and lung samples from the mice in each group were analyzed via flow cytometry on Day 14, and the proportions of various immune cells are shown.

(A) The proportions of the indicated immune cells in the tdLNs of the mice.

(B) The proportions of the indicated immune cells in the lungs of the mice.

The data are presented as the means ± SEMs and were analyzed with an unpaired two-tailed Student's t test. $n = 4$ biological replicates. NS, no significant difference; $^{*}P \leq 0.05$, $^{**}P \leq 0.01$, $^{***}P \leq 0.001$.

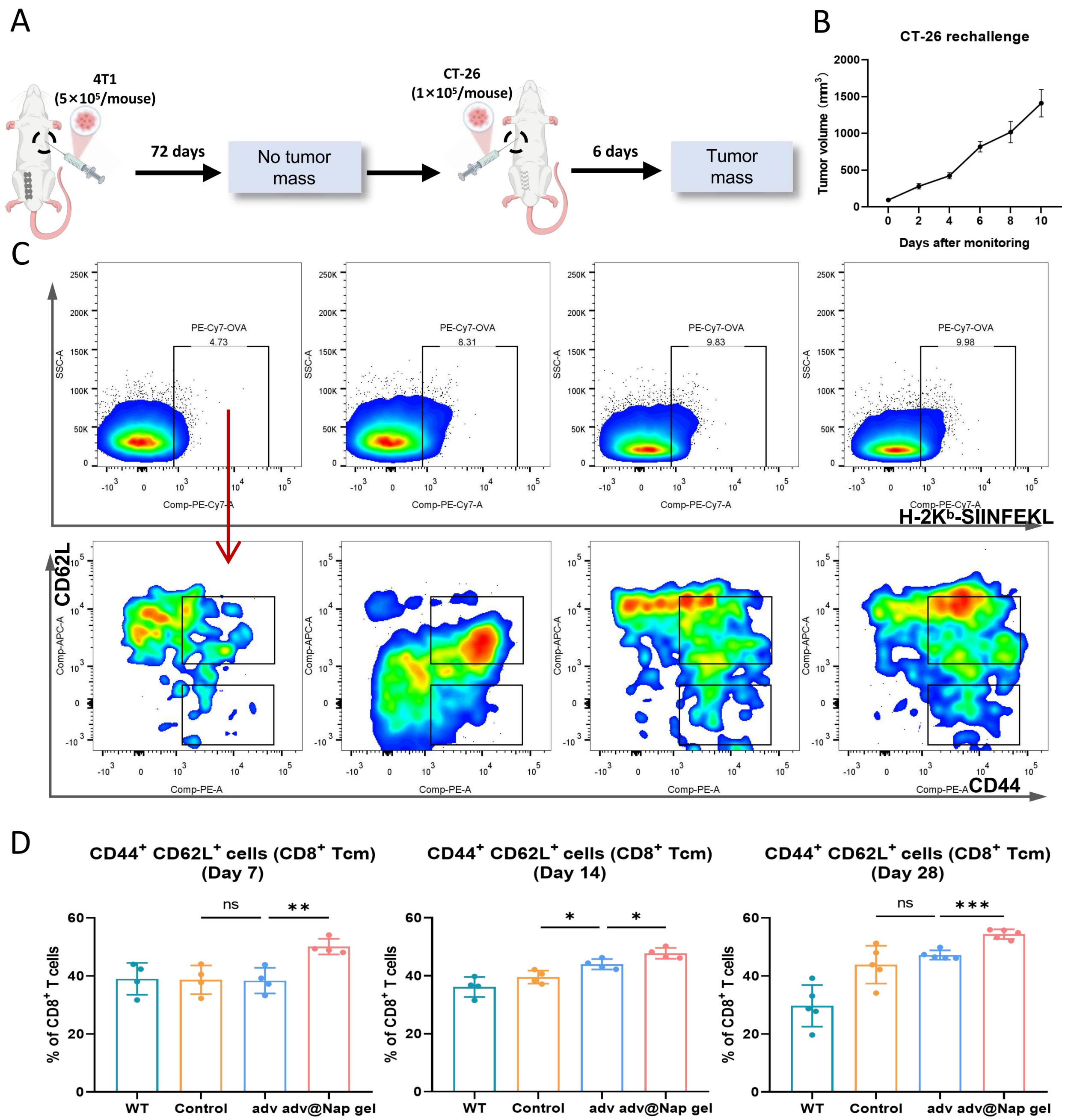

Figure S9. Intraoperative *in situ* treatment with the hydrogel adv@Nap gel induced persistent antigen-specific immune memory. Related to Figure 3.

(A) Schematic illustration of the tumor rechallenge experiment.

(B) Tumor volume after CT-26 rechallenge.

(C) Representative flow cytometric analysis of antigen-specific central memory $CD8^+$ T cells by H-2K^b-SIINFEKL tetramer staining.

(D) Proportion of antigen-specific central memory $CD8^+$ T cells in the spleens of the mice on Day 7, Day 14 and Day 28 after surgery.

The data are presented as the means ± SEMs and were analyzed with an unpaired two-tailed Student's t test. $n = 4\text{-}5$ biological replicates. NS, no significant difference; $*P \leq 0.05$, $**P \leq 0.01$, $***P \leq 0.001$.

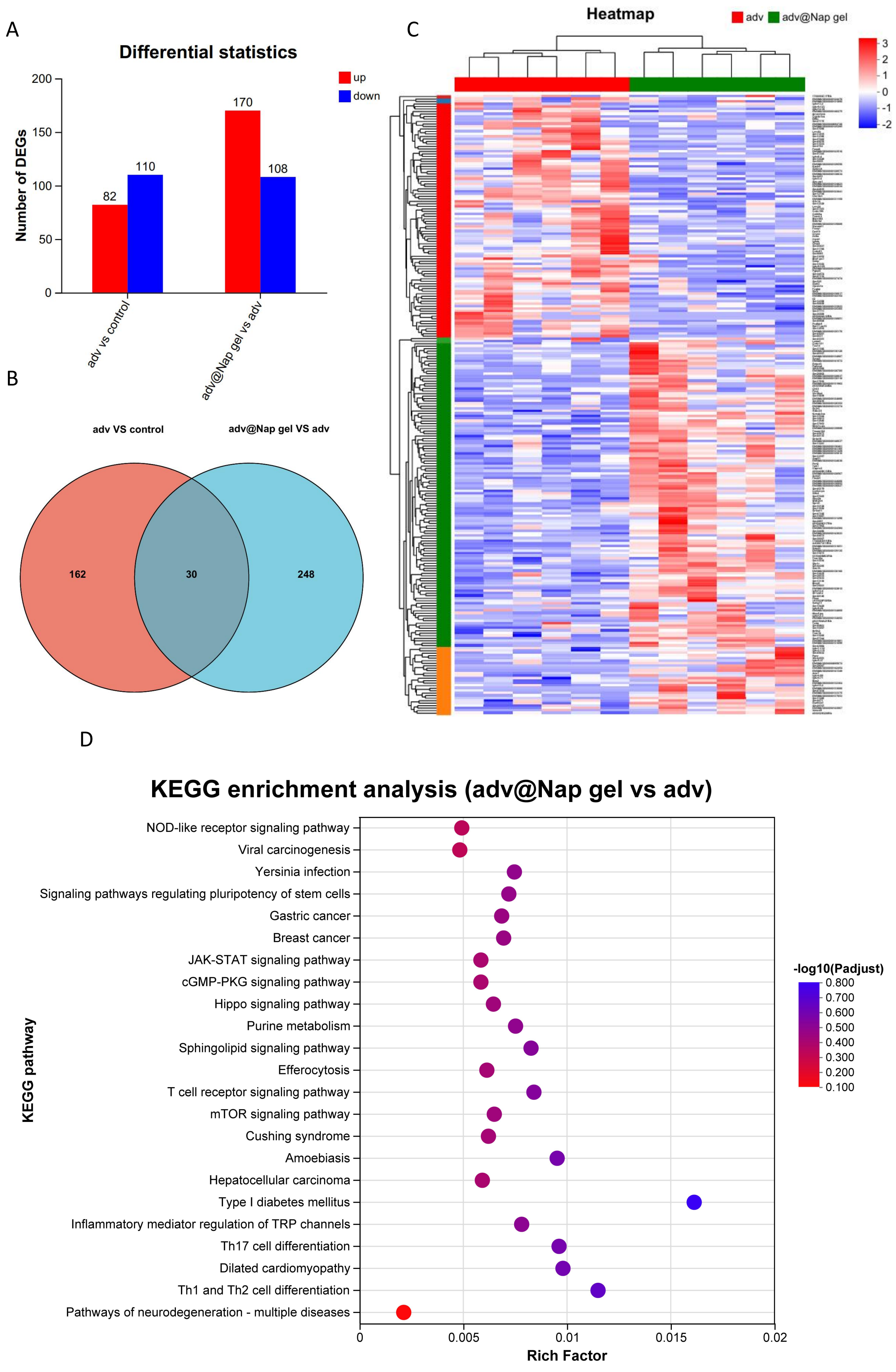
A
Differential statistics
up
down
Number of DEGs
200
150
100
50
0
82
110
170
108
adv vs control
adv@Nap gel vs adv
B
adv VS control
adv@Nap gel VS adv
162
30
248
C
Heatmap
adv
adv@Nap gel
3
2
1
0
-1
-2
D
KEGG enrichment analysis (adv@Nap gel vs adv)
NOD-like receptor signaling pathway
Viral carcinogenesis
Yersinia infection
Signaling pathways regulating pluripotency of stem cells
Gastric cancer
Breast cancer
JAK-STAT signaling pathway
cGMP-PKG signaling pathway
Hippo signaling pathway
Purine metabolism
Sphingolipid signaling pathway
Efferocytosis
T cell receptor signaling pathway
mTOR signaling pathway
Cushing syndrome
Amoebiasis
Hepatocellular carcinoma
Type I diabetes mellitus
Inflammatory mediator regulation of TRP channels
Th17 cell differentiation
Dilated cardiomyopathy
Th1 and Th2 cell differentiation
Pathways of neurodegeneration - multiple diseases
KEGG pathway
0
0.005
0.01
0.015
0.02
Rich Factor
-log10(Padjust)
0.800
0.700
0.600
0.500
0.400
0.300
0.200
0.100

Figure S10. Intraoperative *in situ* treatment with the hydrogel adv@Nap gel induced an obvious shift in the expression profile. Related to Figure 4.

(A) Statistics of the DEGs between the adv group and the control group and between the adv@Nap gel group and the adv group.

(B) Venn analysis of the DEGs between the "adv vs control" counterpart and the "adv@Nap gel vs adv" counterpart.

(C) Heatmap of DEGs between the adv@Nap gel group and the adv group (n = 6 biological replicates).

(D) KEGG enrichment analysis of the target gene set between the adv@Nap gel group and the adv group (n = 6 biological replicates).

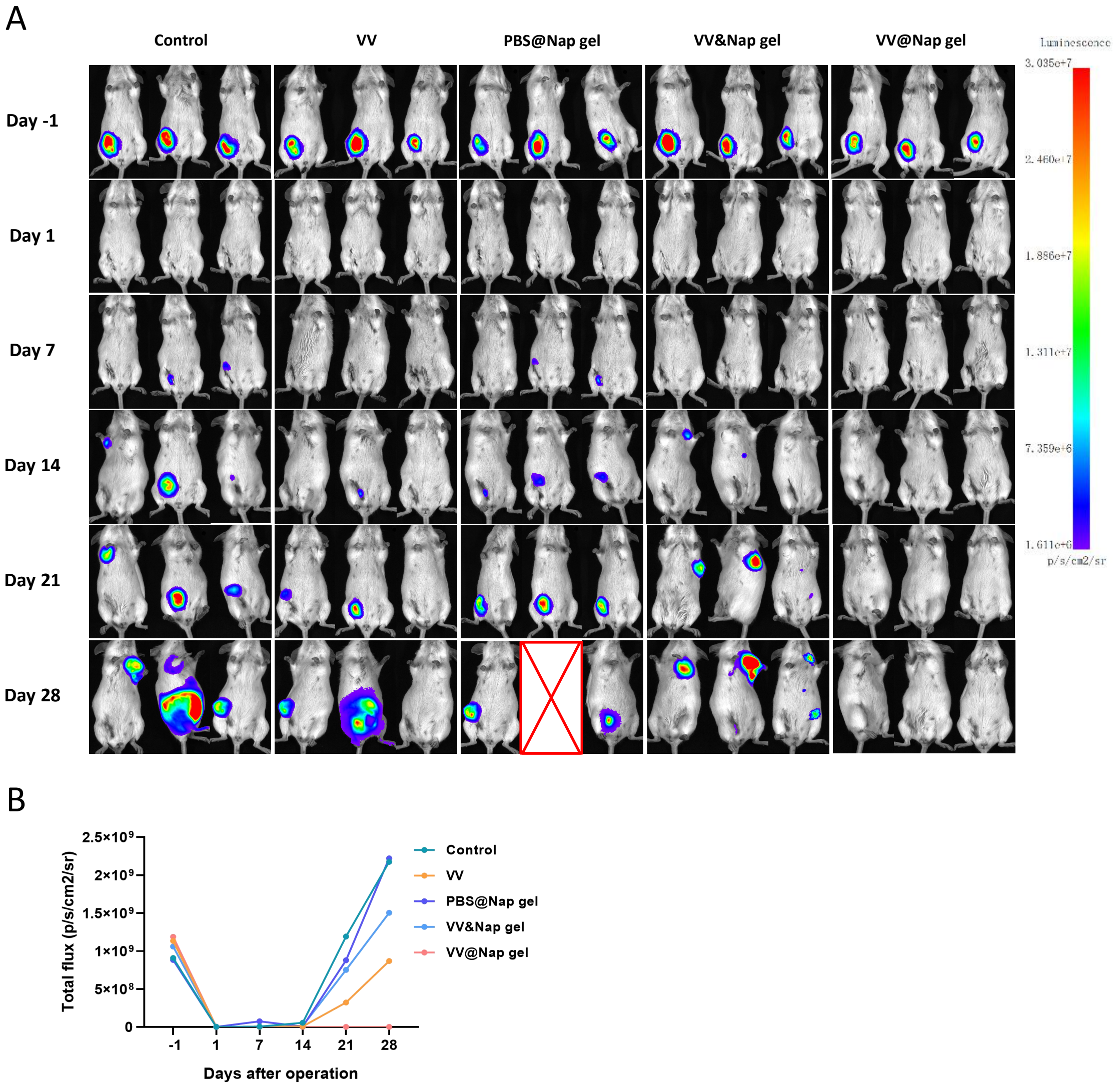

Figure S11. ***In situ*** **treatment with VV@Nap gel instantly after tumor resection controlled tumor recurrence and metastasis. Related to Figure 6.**

(A) Representative IVIS images of 4T1-Luc cells in all groups treated with HSV at different time points after surgery (*n* = 3 biological replicates).

(B) Quantitative statistics of tumor growth in each group according to total fluorescence intensity at the indicated time points (*n* = 3 biological replicates).

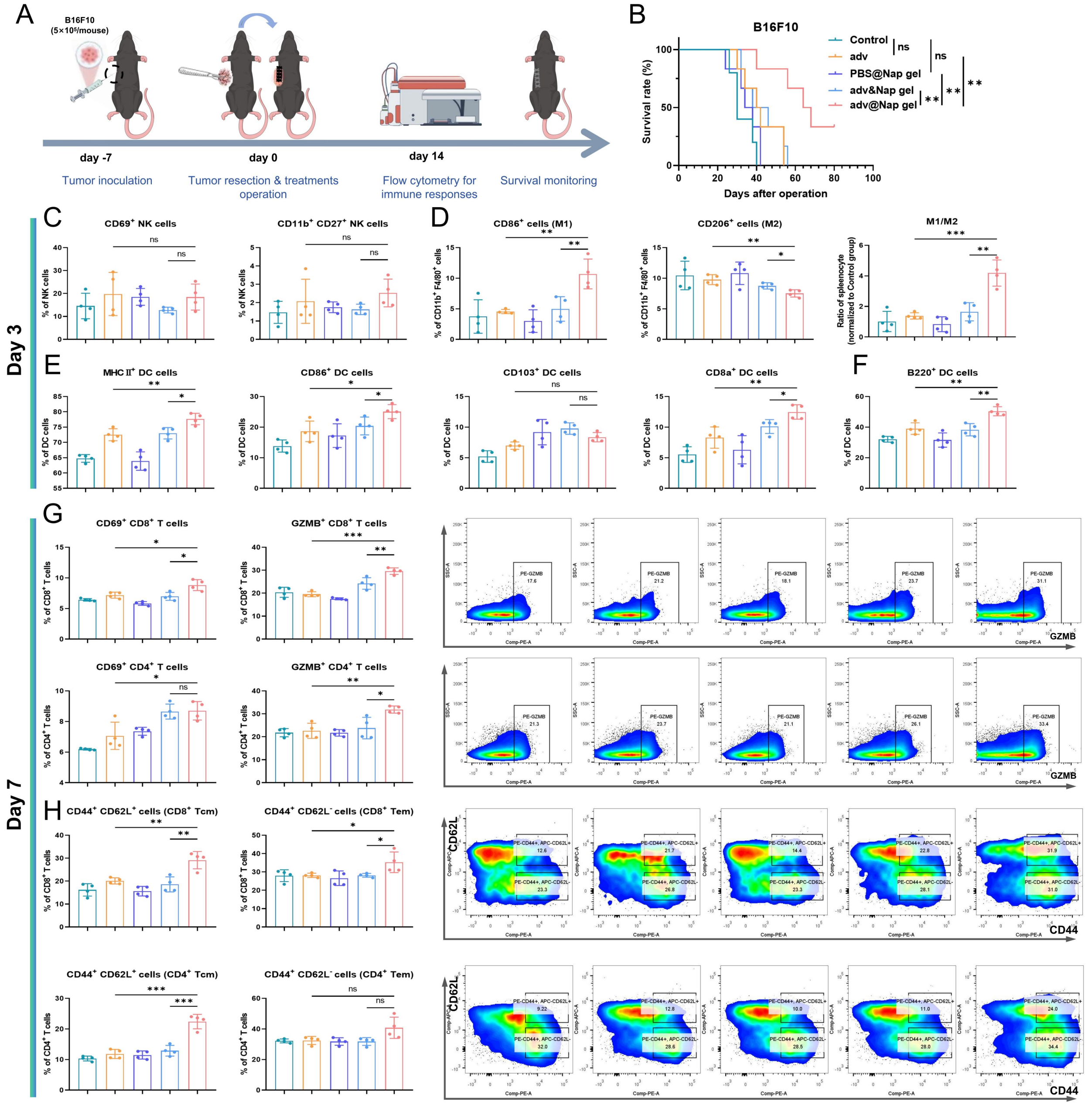

Figure S12. Intraoperative *in situ* treatment with the hydrogel adv@Nap gel controls tumor recurrence in a mouse melanoma model and induces an antitumor immune response. Related to Figure 7.

(A) Schematic illustration of the ability of adv@Nap gel to control tumor recurrence in a mouse model of postoperative melanoma recurrence.

(B) The postoperative survival curves of the mice in each group are shown, and the significance of differences was analyzed (n = 5–6 biological replicates, Kaplan–Meier method with the log rank test).

(C-H) Spleen samples from the mice in each group were analyzed via flow cytometry, and the proportions of various immune cells are shown.

(C) Activated NK cells ($CD69^+$) and high effector NK cells ($CD11b^+$ $CD27^+$) on Day 3.

(D) M1 ($CD86^+$ F4/80^+ $CD11b^+$), M2 ($CD206^+$ F4/80^+ $CD11b^+$) and the M1/M2 ratio on Day 3.

(E) Activated DCs ($MHCII^+$ $CD11c^+$ $CD11b^+$ or $CD86^+$ $CD11c^+$ $CD11b^+$) and DCs that cross-present antigens and generate an antitumor immune response ($CD103^+$ $CD11c^+$ $CD11b^+$ or $CD8\alpha^+$ $CD11c^+$ $CD11b^+$) on Day 7.
(F) Plasmacytoid DCs ($B220^+$ $CD11c^+$ $CD11b^+$) on Day 3.
(G) Activated $CD8^+$ T cells ($CD69^+$), cytotoxic $CD8^+$ T cells ($GZMB^+$) and representative flow cytometry plots, activated $CD4^+$ T cells ($CD69^+$), and cytotoxic $CD4^+$ T cells ($GZMB^+$) and representative flow cytometry plots on Day 7.
(H) Central memory $CD8^+$ T cells ($CD44^+$ $CD62L^+$), effector memory $CD8^+$ T cells ($CD44^+$ $CD62L^-$) and representative flow cytometry plots; central memory $CD4^+$ T cells ($CD44^+$ $CD62L^+$), effector memory $CD4^+$ T cells ($CD44^+$ $CD62L^-$) and representative flow cytometry plots on Day 14.
The data are presented as the means ± SEMs and were analyzed with an unpaired two-tailed Student's t test. $n = 4$ biological replicates; $*P \leq 0.05$, $**P \leq 0.01$, $***P \leq 0.001$.
